# Clandestine nanoelectromechanical tags for identification and authentication

**DOI:** 10.1038/s41378-020-00213-2

**Published:** 2020-11-30

**Authors:** Sushant Rassay, Mehrdad Ramezani, Sumaiya Shomaji, Swarup Bhunia, Roozbeh Tabrizian

**Affiliations:** Electrical and Computer Engineering Department, University of Florida, Gainesville, FL 32611 USA

**Keywords:** Electrical and electronic engineering, NEMS

## Abstract

The realization of truly unclonable identification and authentication tags is the key factor in protecting the global economy from an ever-increasing number of counterfeit attacks. Here, we report on the demonstration of nanoscale tags that exploit the electromechanical spectral signature as a fingerprint that is characterized by inherent randomness in fabrication processing. Benefiting from their ultraminiaturized size and transparent constituents, these clandestine nanoelectromechanical tags provide substantial immunity to physical tampering and cloning. Adaptive algorithms are developed for digital translation of the spectral signature into binary fingerprints. A large set of tags fabricated in the same batch is used to estimate the entropy of the corresponding fingerprints with high accuracy. The tags are also examined under repetitive measurements and temperature variations to verify the consistency of the fingerprints. These experiments highlight the potential of clandestine nanoelectromechanical tags for the realization of secure identification and authentication methodologies applicable to a wide range of products and consumer goods.

## NEMS for security: tamper-proof wireless identification tags at the nanoscale

Nanoscale, tamper-proof product identification tags offer significant benefits over currently used technologies. Conventional product ID tags, such as QR codes or RFID tags, are intrinsically vulnerable to tampering or cloning. Roozbeh Tabrizian, and a team from the University of Florida, United States, created a new ID tag technology based on NEMS (nano-electro-mechanical systems). The NEMS tag is tiny, transparent, and relies on the nanoscale variation in batch-manufactured materials to produce many tags with unique fingerprints. The fingerprints comprise an individual electromagnetic spectra profile, which can be read and converted to digital ID. Due to its small size and transparency, NEMS tags are difficult to differentiate and can be applied to a wide range of products. The team say their tags are easily read in a high-interference environment, and are low cost.

## Introduction

Product counterfeiting, once known as a major infection only in the global economic system, is now escalating, imposing broad social damage and international security threats^1–3^. In addition to influencing legitimate producers by imposing unfair economic competition, counterfeiting is now clearly identified as a reciprocal promoter of organized crime and terrorism^4–6^, forced or child labor^7^, and identity theft and fraud^8^. Product counterfeiting has been conventionally fought using physical tags that enable the identification, authentication, and tracking of genuine items. These tags are identified by designating a digital string to their inherent physical characteristics, hence creating fingerprints (i.e., when the characteristics are unique to each tag) or watermarks (i.e., when the characteristics are identical among a group of tags). The major figures of merit defining the effectiveness of a physical tag are (a) its perseverance to cloning, removal and reapplication, and tampering, (b) its applicability to different goods ranging from electronics to edibles, and (c) the associated cost of production and integration. A wide variety of general-purpose physical tag technologies have been developed, including the Universal Product Code (UPC)^9^, quick response (QR) patterns^10^, and radio-frequency identification (RFID) tags^11^. These technologies suffer from fundamental limitations that make them susceptible to cloning, tampering, distortion, and abolishment. For example, the UPC and QR codes are required to be in the line of sight for optical readout, which makes them easily identifiable for removal and reapplication to counterfeit products. Furthermore, the availability of generators and decoders for UPC and QR barcodes facilitates their imitation at low cost. RFID tags, on the other hand, are based on miniaturized integrated circuits and can store large binary strings, which enhance their resilience to cloning. However, their operation being based on active integrated circuitry and the need for efficient wireless power transfer and interrogation in RFID tags require the integration of large electromagnetic antennas, which makes them inappropriate for clandestine labeling.

Nanoscale physical unclonable functions (nanophysical unclonable functions (PUFs)) have recently emerged to address the substantial limitations of the available identification and authentication tag technologies^12–15^. Nano-PUFs exploit nanofabrication techniques for the miniaturization of static patterns that represent data. While the identification, removal, and reapplication of nano-PUFs impose excessive challenges and require expensive equipment, the operation principle of these tags suffers from similar shortcomings as a result of their macroscale counterparts (i.e., UPC and QR labels), such as the need to be in the line of sight and the unsophisticated information-storage principle.

**Fig. 1:**
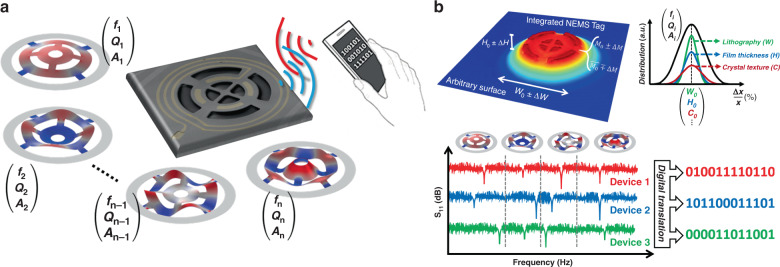
Conceptual demonstration of the NEMS tag concept. **a** a set of mechanical resonance modes with different frequencies (*f*_*i*_), quality factors (*Q*_*i*_), and vibration amplitudes (*A*_*i*_) are excited upon wireless interrogation. The resulting spectral signature is translated into a digital string. **b** The topography of a fabricated NEMS tag, integrated on a glass substrate. The fabrication uncertainties, including film thickness variation, lithographical errors, and randomized crystal polymorphism, induce inhomogeneous variations in the spectral signature of NEMS tags and result in the realization of digital strings unique to each tag

In this paper, we demonstrate a radically different approach based on nanoelectromechanical systems (NEMSs) for the realization of clandestine physical tags with substantial immunity to tampering and cloning and generic applicability to a wide range of products. NEMS tags exploit an electromechanical spectral signature composed of a large set of high-quality-factor (*Q*) resonance peaks; hence, they do not need to be in the line of sight for readout. This principal characteristic, along with their ultraminiaturized size and transparent constituents, makes the clandestine NEMS tags substantially immune to physical tampering and cloning. Furthermore, relying on the large *Q*s of the mechanical resonance modes, which are 2–3 orders of magnitude higher compared to the electrical counterparts used in RFIDs, NEMS tags can be used in cluttered environments with large background noise and interference. Finally, benefiting from the batch fabrication and small footprint, NEMS tags can be manufactured with low cost and used for a wide range of products (see Supplementary Section 1A for a detailed comparison of the NEMS tag technology with other state-of-the-art counterparts). Figure 1 illustrates the NEMS tag concept.

In this work, the design and implementation of clandestine NEMS tags are presented, and their operation principle based on the use of electromechanical spectral signatures along with mathematical algorithms for digital translation is discussed. We further present statistical procedures for exploring the entropy, consistency, and temperature stability of the corresponding digital signatures, and discuss the potential of NEMS for the realization of a new class of physical tags for generic and secure identification and authentication applications.

### Fabrication process

**Fig. 2:**
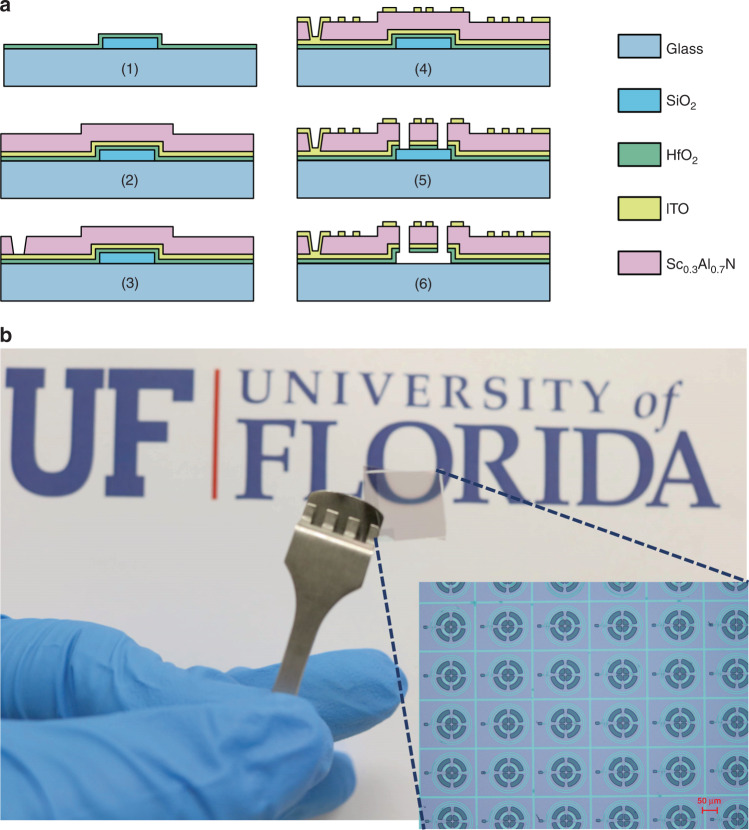
Fabrication of the clandestine NEMS tags. **a** the fabrication process for the implementation of clandestine NEMS tags on a glass substrate: (1) deposition and patterning of a sacrificial SiO_2_ layer on the glass substrate and the ALD of 10-nm HfO_2_, (2) sputtering and patterning of 50-nm ITO (bottom electrode) and 100-nm Sc_0.3_Al_0.7_N, (3) patterning of the Sc_0.3_Al_0.7_N layer to access the bottom ITO electrode, (4) deposition and patterning of the top ITO electrodes and the coil, (5) etching of trenches in the transducer stack to define the NEMS tag geometry, and (6) releasing of the NEMS tag by etching sacrificial SiO_2_. **b** A 1-cm×1-cm glass substrate with very-large-scale-integrated array of NEMS tags featuring optical transparency. The inset shows an enlargement of the optical image, highlighting an array of NEMS tags with identical layouts

A NEMS tag is created by sandwiching a thin piezoelectric film between two metallic layers. To enhance the clandestine attribute of the tag, the constituent layers are chosen among transparent materials. Furthermore, the tags are implemented on a glass substrate to facilitate the evaluation of their transparency. Figure 2a shows the fabrication process used for implementation of the NEMS tags.

**Fig. 3:**
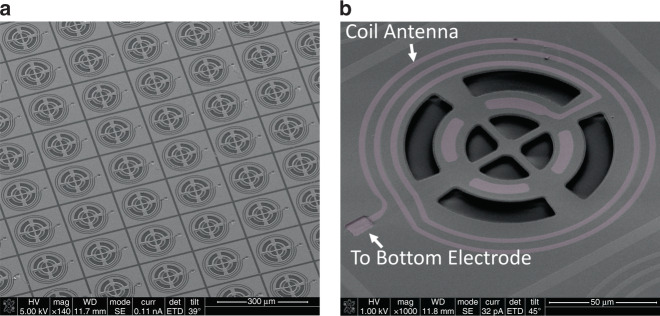
SEM images of the clandestine NEMS tags. **a** an array of NEMS tags with the same layouts implemented in the same batch on a glass substrate; **b** an individual NEMS tag with an integrated coil antenna enabling wireless interrogation of the spectral signature through magnetic coupling

A sacrificial silicon dioxide layer (SiO_2_) is deposited onto the glass substrate. This is followed by atomic layer deposition of a 10-nm-thick hafnia to protect the consequent layers at the final release step. Next, a 50-nm-thick layer of transparent indium tin oxide (ITO) is sputtered to serve as the bottom electrode. The ITO sputtering process is engineered to enhance the electric conductivity and optical transparency (see Supplementary Section 1B for further details). In the next step, a 100-nm-thick piezoelectric scandium-doped AlN (i.e., Sc_0.3_Al_0.7_N) is deposited, followed by deposition and patterning of the second 50-nm-thick ITO layer, to serve as the top electrode. Along with optical transparency, Sc_0.3_Al_0.7_N provides a large electromechanical coupling coefficient, which enables efficient excitation of the mechanical resonance modes with minuscule electromagnetic powers^12^. Finally, the NEMS tag is patterned by etching the transducer stack and released by etching sacrificial SiO_2_ in hydrofluoric acid. Figure 2b shows the glass substrate with a very-large-scale-integrated array of clandestine NEMS tags, highlighting the optical transparency. Figure 3 shows SEM images of the NEMS tags.

### Operating principle

**Fig. 4:**
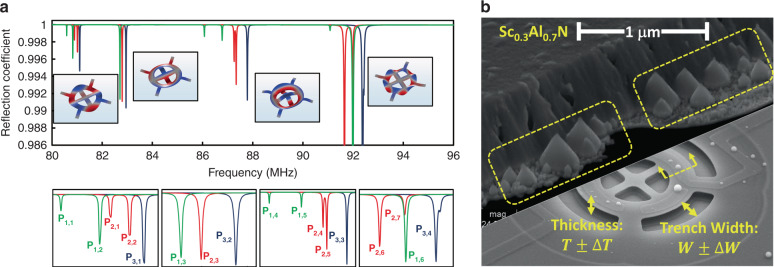
Simulation of NEMS tag spectral signature subjected to randomized structural variations. **a** the large-span simulated spectral signature of the NEMS tags, with randomized variations in their thickness, lateral dimension, and crystalline profiles, and the short-span signature over each resonance peak in the spectral response, highlighting the effect of the nanofabrication uncertainties. **b** An SEM image of the NEMS tag cross section, highlighting the fact that the cubic cones formed randomly during the Sc_0.3_Al_0.7_N growth

The clandestine NEMS tags exploit the electromechanical spectral signature as the physical characteristic for identification. The lateral geometry of the NEMS tag is designed to ensure the creation of a large set of high-*Q* mechanical resonance modes within a frequency range of interest. Figure 4a shows the simulated spectral signature of the NEMS tag, highlighting the large number of high-*Q* resonance peaks within a small frequency range over 80–96 MHz. Different characteristics of the corresponding peaks to these resonance modes can be used to assign a binary string to the NEMS tag. These characteristics include the frequency, *Q* and amplitude of the peaks. All these characteristics are highly sensitive to the uncertainties during fabrication processing of the NEMS tags. Due to their random distribution, fabrication uncertainties enable the natural creation of visually identical NEMS tags with unique and distinct fingerprints within a single batch. While this distinction cannot be identified visually and is reflected only in the spectral signature, reverse engineering of the NEMS tag is very challenging, if not impossible.

The fabrication uncertainties that account for the creation of distinct fingerprints are due to variations in device implementation (i.e., extrinsic) or inherent to the material properties (i.e., intrinsic). Extrinsic uncertainties include thickness variation of the constituent films, lithography errors in patterning electrodes, and device lateral geometry. Intrinsic uncertainties correspond to the variations in material properties that affect the spectral signature of the NEMS tag. These include the elastic constants, mass density, and crystal morphology of the constituent films. While the extrinsic uncertainties depend on the manufacturing facility used for implementation of the tag, intrinsic uncertainties are inherent to the materials and define the ultimate entropy of the labels extracted for each tag.

Ideally, the randomness induced by intrinsic uncertainties in the label constituents is desirable, as it provides two distinct security benefits. First, it allows the creation of unique identifiers (called “fingerprints”) for each instance of millions of batch-fabricated devices that use the same layout. Second, in most practical scenarios, devices are manufactured in untrusted foundries, where an attacker can gather knowledge of the layouts and can potentially create counterfeit products with cloned labels. Therefore, the material-based intrinsic randomness is superior compared to the layout-based approaches. Furthermore, although purposeful layout variations can help enhance the entropy of the tags, they add additional steps to the implementation of the labels and increase their cost.

In this work, the Sc_0.3_Al_0.7_N film used for implementation of the NEMS tags offers large intrinsic uncertainties due to the purely randomized formation of cubic conical clusters within the hexagonal grains^16,17^. This inherent characteristic corresponds to the tendency of the film morphology transition from hexagonal aluminum nitride (AlN) to cubic scandium nitride at high concentrations of scandium doping. Figure 4b shows the cubic conical clusters that emerged after etching the hexagonal grains of Sc_0.3_Al_0.7_N. Benefiting from the ultraminiaturized size of the NEMS tags, the purely random distribution of these clusters within the film translates to a large entropy of the corresponding binary string.

Figure 4a shows the simulated spectral response for tags with identical layouts but randomized intrinsic and extrinsic structural characteristics. Three random structures (*S*_random_,_*i*_,*i* = 1,2,3) are generated to mimic the lithographical uncertainty (*W*_random_,_*i*_), the Sc_0.3_Al_0.7_N film thickness variation (*T*_random_,_*i*_), and the randomized distribution and placement of the conical cubic micrograins within the film (*D*_random_,_*i*_). A random generator is developed in MATLAB to identify the values of *W*_random_,_*i*_ and *T*_random_,_*i*_ over ±10% uncertainty. *D*_random_,_*i*_ is generated through random identification of the position of cubic micrograins over the device area, with a distribution of ten 1-μm^2^ micrograins per 100-μm^2^ area. Three structures are generated with *S*_random_,_*i*_ = {*W*_random_,_*i*_, *T*_random_,_*i*_, *D*_random_,_*i*_} (*i* = 1,2,3), and their spectral signature is simulated using COMSOL. A large variation in the spectral signature is observed, including the change in the number of resonance dips and their frequency and magnitude.

### Translation procedure

**Fig. 5:**
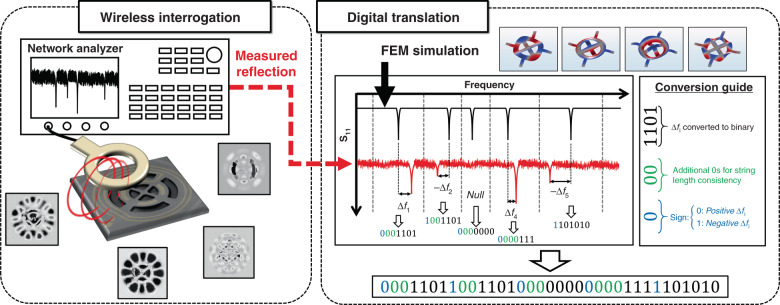
Schematic diagram of the digital translation procedure used to designate unique binary tags to the NEMS labels: the measured spectral signature of a tag is compared with the reference signature extracted from COMSOL simulations. The reference signature is divided into intervals with borders defined by the average of the frequencies of adjacent peaks. In each interval, the measured peak with the highest magnitude is identified, and its frequency is subtracted from the reference peak. The resulting decimal value is converted to a binary substring. A conversion guide is used to assign the leftmost bit to the sign of the subtraction, additional zeros to ensure consistent length of the substrings, regardless of the relative frequency offset of the measurements and reference in each interval, and all zeros when no measured peak exists in an interval. Finally, the substrings are cascaded to create the designated binary tag for the NEMS label

The translation procedure for the generation of the binary strings corresponding to the NEMS tags relies on identification of the resonance peaks within the spectral signature and the use of their frequency. The procedure starts with the identification of a master spectral signature extracted from simulations or an arbitrary tag. This master signature is then used as a reference to extract the decimal frequency mismatch of all the peaks within the signature of the other tags. The resulting decimal numbers, assigned to the peaks, are then converted to binary and cascaded to form the initial string assigned to each tag. Finally, the length of the extracted binary per resonance dip is adjusted to ensure consistency, regardless of the presence of a dip in an interval or its frequency offset from the reference. Figure 5 demonstrates the digital translation procedure, where the measured spectral response of a label is compared with the reference spectral signature generated by COMSOL simulations. The comparison intervals are defined by identification of the boundaries at the average frequency of the two adjacent peaks in the simulated response. In each interval, the measured spectral response of a label is monitored to identify the dip with the highest magnitude. The frequency of this dip is then subtracted from its counterpart in the simulated reference signature. The resulting decimal value is converted to a binary string, where the leftmost bit defines the sign. Additional bits with 0 values are added to ensure a constant length of the binary string in each interval, regardless of the frequency offset between the simulated and measured values (see Supplementary Section 1C for further details).

### NEMS tag characterization and statistical discussions

The spectral signatures of the NEMS tags are measured using near-field wireless interrogation over the frequency span of 80–90 MHz. An ICR magnetic near-field microprobe, with a coil radius of 50 μm, is positioned atop the NEMS tag to enable wireless interrogation through magnetic coupling. The microprobe is positioned at a sub-2-mm vertical distance from the label and connected to a network analyzer to enable measurement of the reflection response (i.e., S_11_) over the 80–90-MHz spectrum. Prior to the measurement, a calibration procedure is carried out to de-embed the impedance-loading effect of the microprobe on the spectral response.

**Fig. 6:**
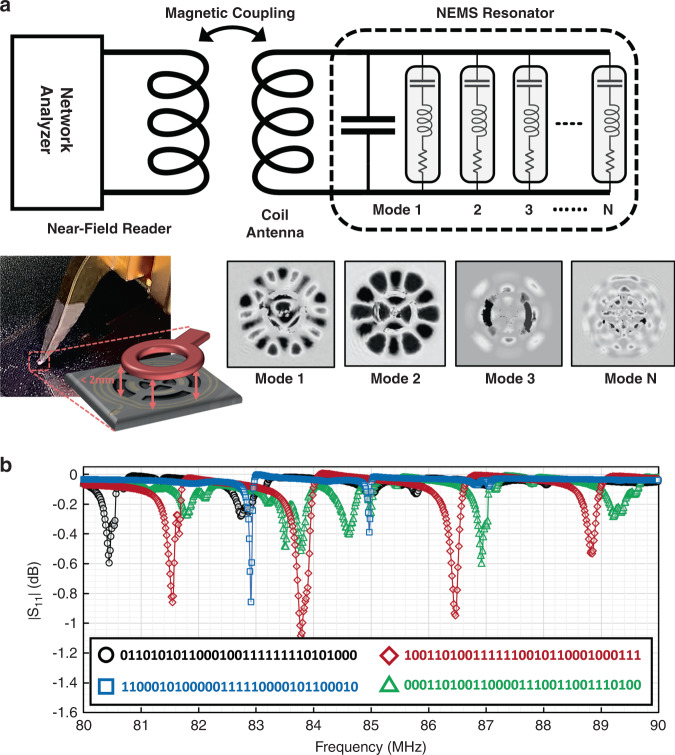
Wireless spectral interrogation of the NEMS tags. **a** the near-field wireless interrogation setup used for extraction of the spectral signature of the NEMS tags. The inset shows various mechanical vibration patterns corresponding to resonance modes in the spectral signature, measured by the holographic microscope. **b** The measured spectral signature of three NEMS tags with identical designs and fabricated in the same batch. The inset shows the 31-bit binary strings extracted for each tag

Figure 6a shows the wireless interrogation setup and a representative model of a NEMS tag, composed of an integrated coil antenna, a capacitor representing the piezoelectric transducer, and N mechanical RLC motional branches corresponding to different resonance modes. In Fig. 6a, the inset shows the optical image of the device under test, as well as the corresponding vibration patterns at different resonance frequencies extracted using optical probing (see Supplementary Section 2 for live-vibration mode-shape videos of different modes, captured using a holographic microscope). To enhance the wireless interrogation resolution, the static response corresponding to the electrical feedthrough induced by the magnetic coupling coils and piezoelectric film capacitor is de-embedded using nonreleased NEMS tags. Figure 6b compares the measured spectral signatures of four NEMS labels that are randomly picked from the array shown in Fig. 3. The significant distinction between the vibration patterns corresponds to the different surface and bulk acoustic waves that create the resonance modes. This distinction is responsible for the highly randomized effect of intrinsic and extrinsic uncertainties on the spectral signature of the NEMS tags. Figure 6b also shows the corresponding binary labels for the four tags extracted using the digital translation procedure. The 31-bit string assigned to the spectral signature fingerprints highlights the large entropy of the clandestine NEMS technology, which makes it suitable for identification, authentication, and tracking applications.

**Fig. 7:**
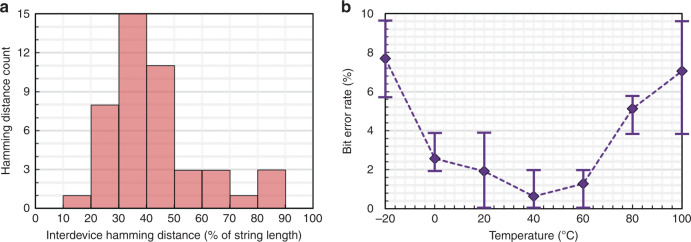
Entropy and robustness characterization of the NEMS tags: **a** the extracted histogram for the interdevice Hamming distance for a set of ten NEMS tags with identical design. **b** The bit-error rate for the ten NEMS tags, extracted from −20 to 100°C, over three consecutive cycles

To quantify the entropy, ten NEMS tags with identical designs are used. The interdevice Hamming distance is used as a metric to measure the uniqueness of the binary strings corresponding to the spectral signatures^18^. Figure 7a shows a histogram of the interdevice Hamming distance extracted from a group of ten identical NEMS tags. The tags are also measured across temperature variations to identify their environmental robustness. The temperature sensitivity of the material properties and device dimensions results in finite variation of the frequency of the mechanical resonance modes. To quantify the effect of the temperature variation on the extracted binary strings, the ten NEMS tags are measured over −20 °C to 100 °C in 20 °C steps, and their corresponding binary strings are compared to extract the bit-error rate (BER). Temperature characterization is performed on a single device and in three consecutive cycles to further evaluate the robustness of the binary tags over repeated measurements. Figure 7b shows the average BER at different temperatures. The error bar at each data point shows the distribution of the BER over the three measurement cycles. A BER lower than ~8% is measured over −20 to 100 °C, highlighting the temperature stability of the tags for practical applications. Furthermore, the BER variation across measurement repetitions is smaller than ±3% over the entire temperature range, which indicates the robustness of the NEMS tags. The temperature stability, despite the sensitivity of the resonance frequencies, is attributed to the consistent temperature coefficient of frequency (TCF) of the peaks (i.e., −32 ppm/°C < TCF < −34 ppm/°C) across the entire group of NEMS tags. This results in an equal fractional shift in the frequency of the peaks, which does not affect the extracted binary string considering the spectral normalization used in the digital translation procedure.

## Conclusions

In conclusion, we have demonstrated a new physical tag technology for identification and authentication that is based on the use of the electromechanical spectral signature of clandestine NEMSs. The clandestine NEMS tags are created by patterning a thin transducer stack formed from transparent electrodes and a piezoelectric layer and are accommodated with an integrated coil antenna that enables near-field wireless interrogation. The spectral signature of the NEMS tag is engineered to have a large number of high-*Q* mechanical resonance peaks with frequencies and amplitudes that are randomized by fabrication uncertainties. Intrinsic variations in the material properties and extrinsic disparities in the lithography and deposition steps used to create the NEMS tag result in unique spectral signatures that designate distinct fingerprints. A translation algorithm is used to designate a binary string to the spectral signature of each tag. NEMS tags are fabricated using Sc_0.3_Al_0.7_N films sandwiched between ITO electrodes and show up to 8 high-*Q* resonance modes over the 80–90-MHz frequency range. This spectral signature corresponds to a 31-bit binary string. The large entropy and robustness of the NEMS tags highlights the ability of this technology to identify and authenticate products and goods in large supply. Benefiting from their ultraminiaturized size, optical transparency, and visually undetectable and indirect principles of information storage, the clandestine NEMS tags offer a transforming identification and authentication approach to protect the supply chain against tampering and cloning attacks that target product counterfeiting.

## Methods

### Device fabrication

The NEMS tags are fabricated by various etching and deposition processes to create free-standing devices and enable electrical pads for the application of excitation and sensing signals (see Supplementary Section 1B for details).

### Device characterization

The NEMS tags are electrically characterized using an ICR HH 100-27 Magnetic Near-Field Probe sold by Langer EMV-Technik and a Keysight N5222A network analyzer to extract the spectral signature. Optical characterization for the extraction of vibration patterns is performed using a Lyncée tec R-2100 series reflection digital holographic microscope.

### Scanning electron microscopy

SEM images are taken using an FEI Nova NanoSEM 430 system.

## Supplementary information

Supplementary Information 1

Supplementary Information 2

## Data Availability

The authors declare that the main data supporting the findings of this study are available within the article and its Supporting Information files. Extra data are available from the corresponding author upon request.
