# Supplementary material for: Clandestine nanoelectromechanical tags for identification and authentication
Source: Microsyst Nanoeng. 2020 Nov 30;6:103. doi: 10.1038/s41378-020-00213-2 (PMC8433297; doi:10.1038/s41378-020-00213-2)
Supplement: Supplementary file 1 — Supplementary Information 1 [file 41378_2020_213_MOESM1_ESM.pdf]

SECTION 1

A) Comparison with State-of-the-Art Identification Tag Technologies

Table 1 compares the presented resonant NEMS labels with other tag technologies used for identification and authentication. Different figures of merits are considered for the comparison.

	**QR Code**	**RFID**	**Resonant M/NEMS Label**
**Size**	***Minimum size:*** • 21 modules by 21 modules (dots) ***Maximum size:*** • 177 modules x 177 modules (dots)	***IC Minimum size:*** • 0.15mm x 0.15mm (smallest recorded size by Hitachi)	***Minimum Size:*** • Nano-meter scaling (sub 100-nm)
**Invisibility**	• Cannot be invisible due to sizing requirements for QR code dots for functionality.	• Visible due to the RF antenna and actual "tag" the microchip sits on.	• Not visible to the human eye without mechanical assistance.
**Entropy**	• 40 possible QR Code versions ranging from a minimum of 7 binary bits and a maximum of 2,953 binary bits	• Typically stores 96-512 bits of memory	***MEMS prototypes:*** • Entropy can be set based on frequency range set for device.
**Cost**	• Price based on the QR Code generator used. • Estimated $5/QR Code • QR code scanner prices range from $40-$300	• Ranges from $0.05 to $50, depending on the application. • RFID Tag scanner prices range from $500- $2,000	***Envisioned*** • $0.01-$0.05 benefiting from wafer-level batch fabrication / miniaturized size.
**Robustness**	• Can be used for different applications. • Error code correction allows functionality with some damage and distortion to QR Code. • Readable from all directions.	• Cannot be used on all materials such as: liquid and metal products. • Environmental variations (such as sensor signal clutters, temperature, etc.) can cause dysfunctionality.	• Physical and variations (i.e., electrode positioning and number of nanodots, temperature, power) will not distort digital signature • Ability to use on most material platforms
**Vulnerability to Tampering**	• Can be decoded using QR decoder techniques found via internet. • No true security • Easy malicious manipulation by hackers.	• Can be duplicated using reverse engineering techniques • Easily damaged or destroyed • Easily removed	• Clandestine (visually untraceable) • If detected, intrusive tampering will damage device / not allow for reverse engineering. • Vulnerability VERY limited due to size.
**Application Space**	• Consumer goods • Retail • Entertainment (i.e., concert and sports tickets) • Electronics • Social Applications	• Retail • Access management (i.e., hotel rooms, ID badges) • Military • Transportation • Toll collection • Government documents (i.e., passports) • Human Implantation	***Envisioned:*** • Consumer goods • Retail • Entertainment • Electronics • Currency • Government documents • Access management • Military Applications

B) Transparent ITO Electrode Fabrication:

ITO thin films were deposited at room temperature on a glass/SiO_2/$Hf0_2$/$Sc_{0.3}Al_{0.7}N$ substrate by the Kurt J. Lesker Multi-Source RF and DC Sputter System. The ITO sputtering target provided by Kurt J. Lesker company consists of In_2O_3/SnO_2, which is 90/10 in percentage by weight. The conductivity and transparency of the ITO film are a function of the gas types and flow rates as well as the DC bias voltage. Several experiments are carried out to maximize the transparency and conductivity of the film, which is concluded in the optimum recipe with 35 sccm flow of Argon (no additional oxygen), RF/DC powers of 125/20 Watts under a chamber pressure of 3.9e-3 Torr. The deposition rate was 0.87 Å/sec. The VEECO FPP-5000 four-point probe was used to measure the resistivity of the sputtered ITO film, which resulted in 9.5e-4 ohm.cm.

C) Digital Translation Procedure:

The following procedure is used to generate the unique binary string designated to each NEMS tag:

1. Chose an arbitrary tag to serve as the reference for generation of binary labels for a group of NEMS tags.
2. Adjust the spectral signature of other tags, by scaling their frequency, to have the frequency of their first peak matching the with the first peak of the reference tag.
3. Define frequency intervals in the spectral signature of the reference tag so that each interval contain a peak.
4. Use these intervals to locate resonance peaks in other tags scaled spectrum.
5. If an interval contains several peak, choose the one with largest magnitude.
6. Find the decimal difference between the frequency of the peak in each interval with corresponding peak in reference tag.
7. Generate a binary number out of the decimal values extracted in previous step. Assign a first bit to identify the sign of frequency difference (0 when positive; 1 when negative).
8. In reference tag’s spectral signature: For each interval, define the maximum length of the corresponding binary string through identifying the frequency offset of the peak with either ends of the interval.
9. Add 0s to the binary string corresponding to each interval, in non-reference tags, to ensure identical string length for all the tags.
10. Cascade the binary strings generated for each interval to form the final digital label for each NEMS tag.
