# Supplementary material for: Clandestine nanoelectromechanical tags for identification and authentication
Source: Microsyst Nanoeng. 2020 Nov 30;6:103. doi: 10.1038/s41378-020-00213-2 (PMC8433297; doi:10.1038/s41378-020-00213-2)
Supplement: Supplementary file 2 — Supplementary Information 2 [file 41378_2020_213_MOESM2_ESM.pptx]

## Slide 1
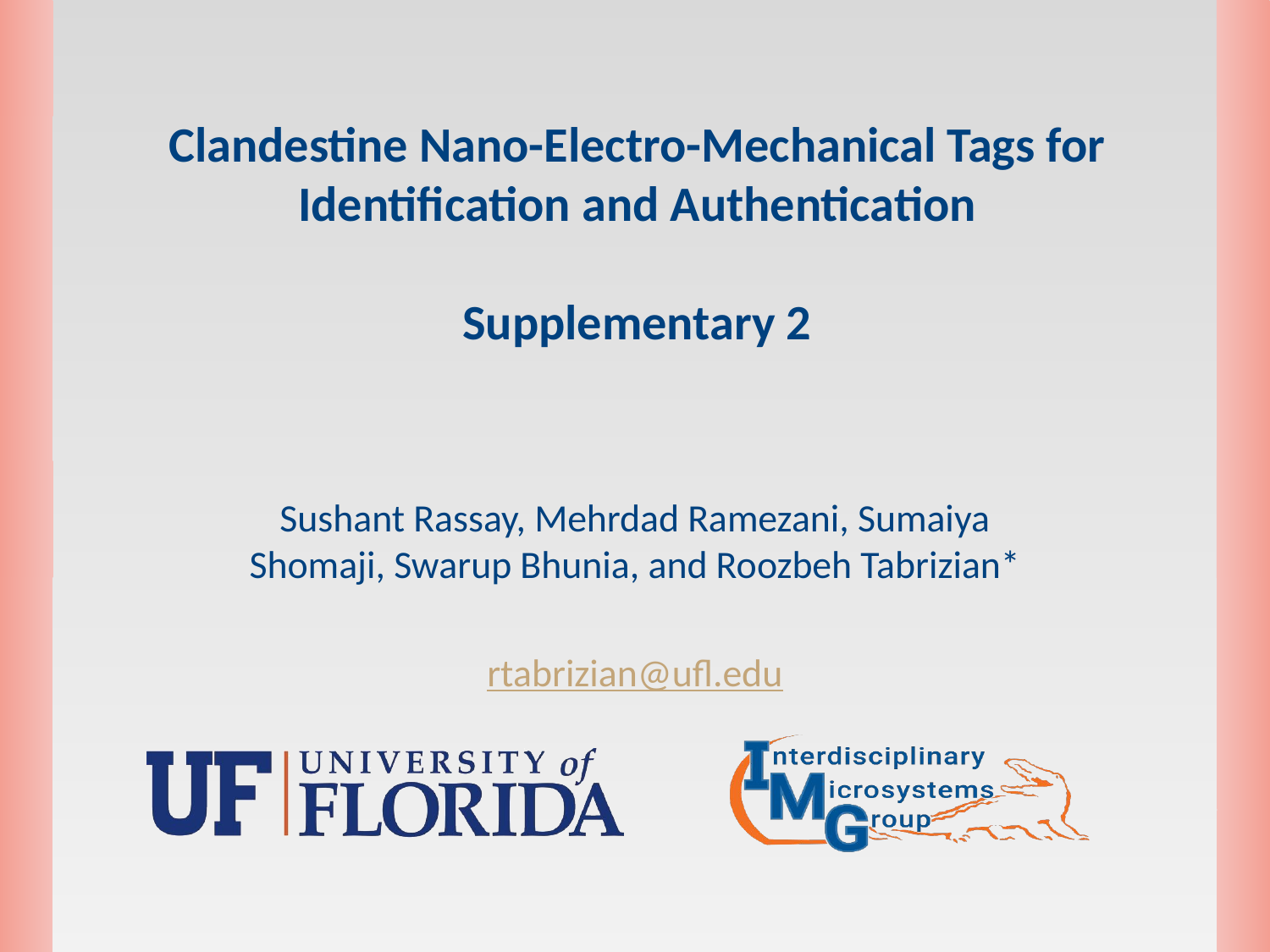

# Clandestine Nano-Electro-Mechanical Tags for Identification and Authentication Supplementary 2
Sushant Rassay, Mehrdad Ramezani, Sumaiya Shomaji, Swarup Bhunia, and Roozbeh Tabrizian*
rtabrizian@ufl.edu

## Slide 2
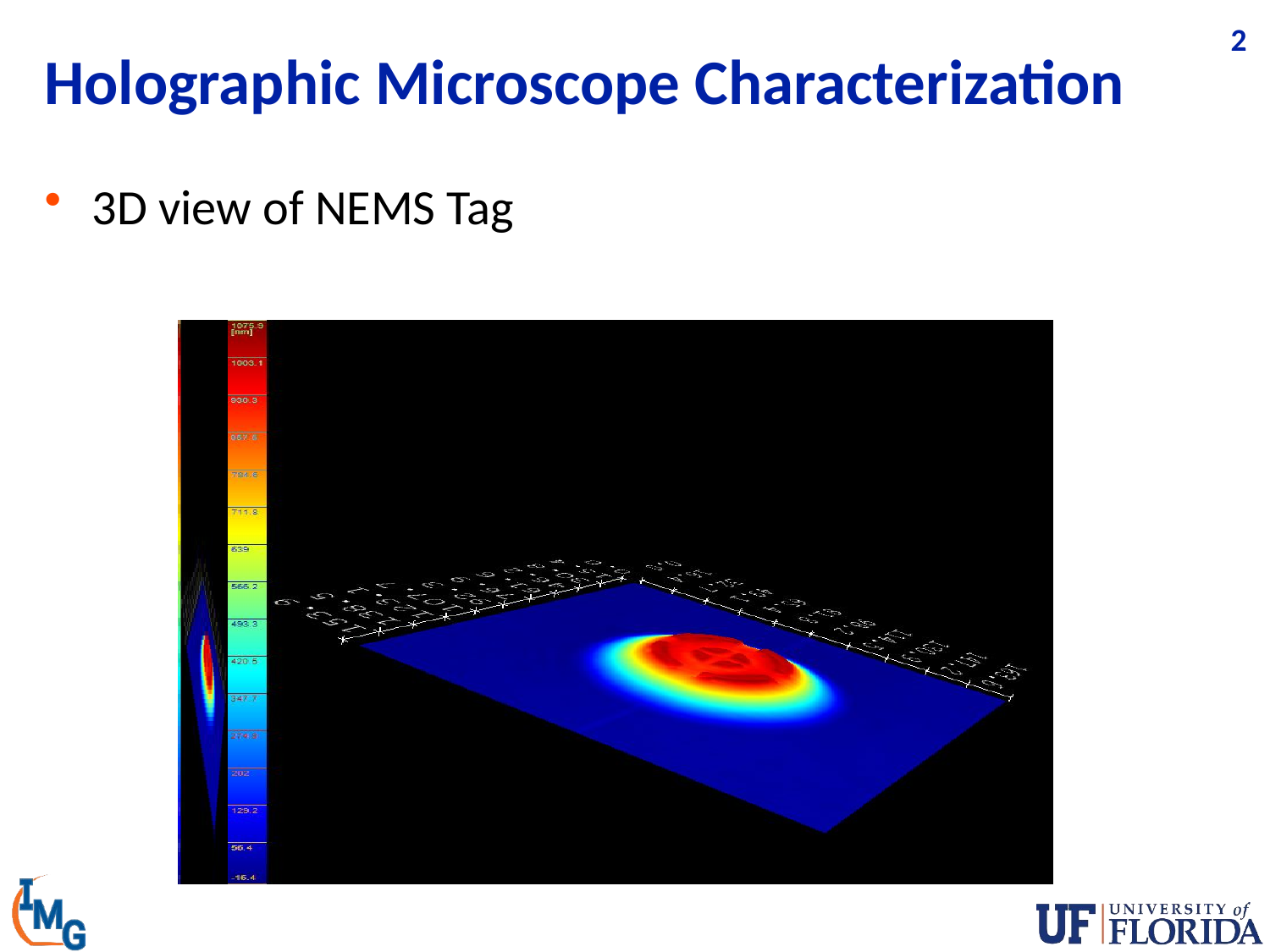

# Holographic Microscope Characterization
1
3D view of NEMS Tag

## Slide 3
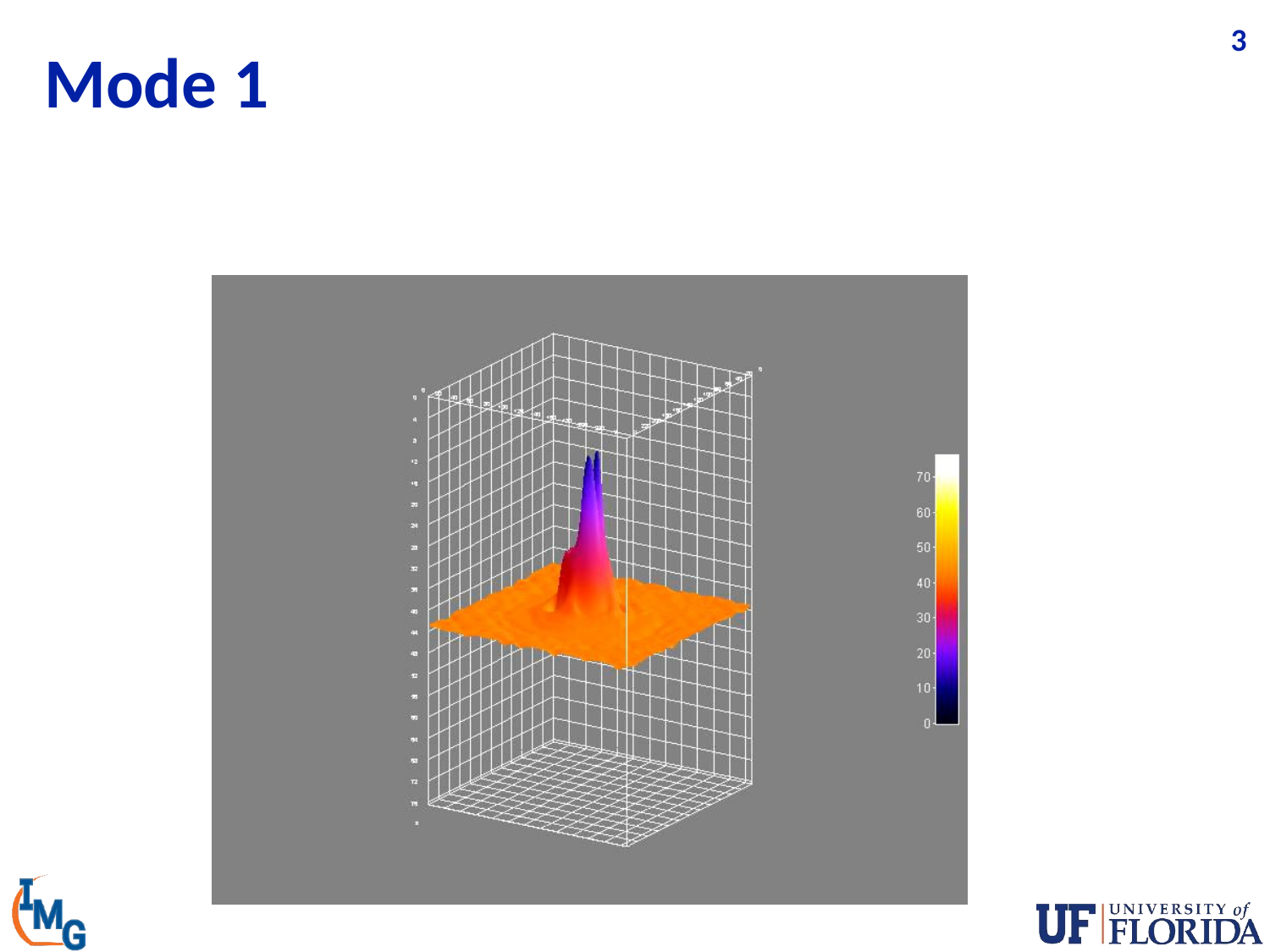

# Mode 1
2

## Slide 4
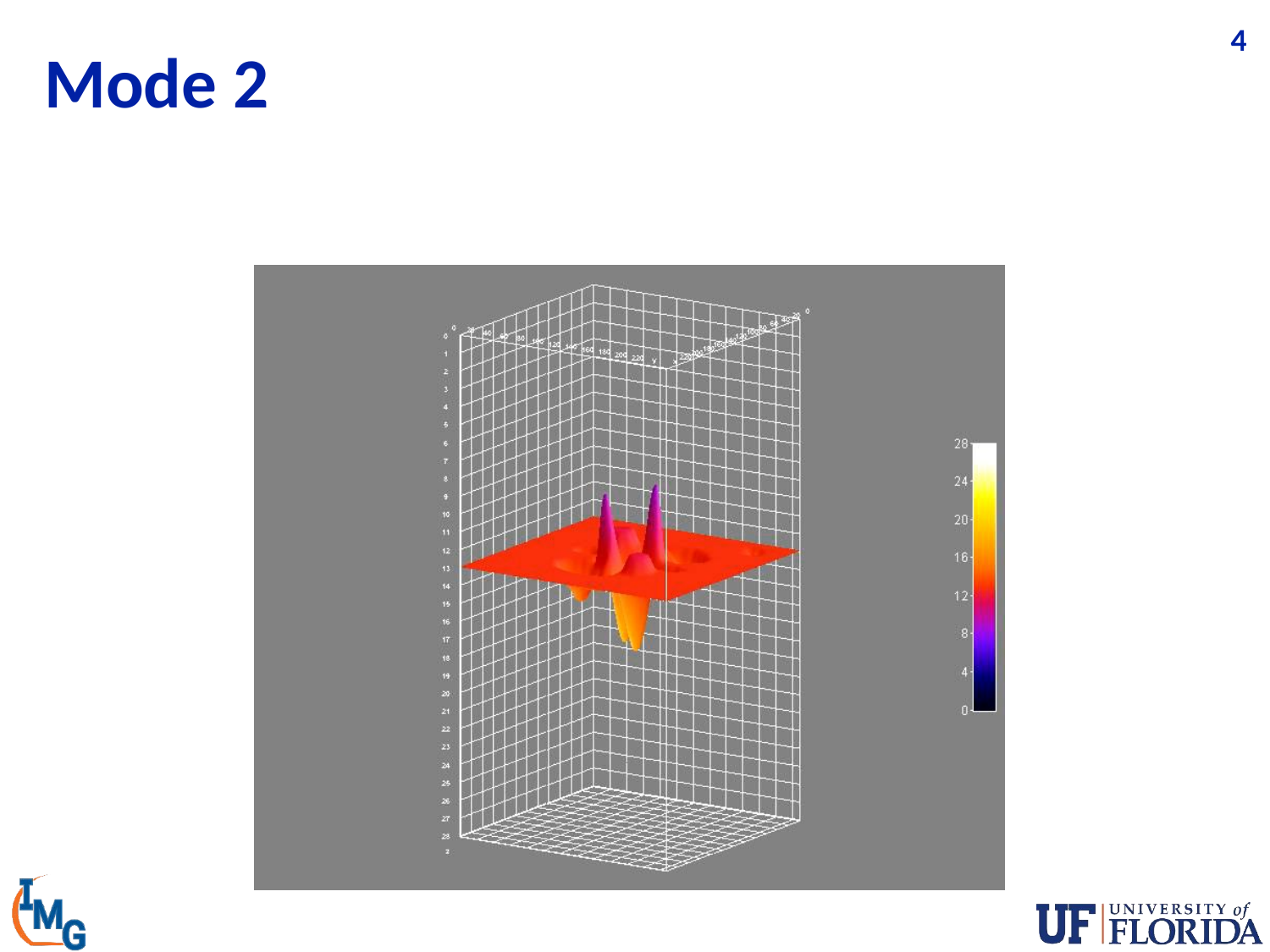

# Mode 2
3

## Slide 5
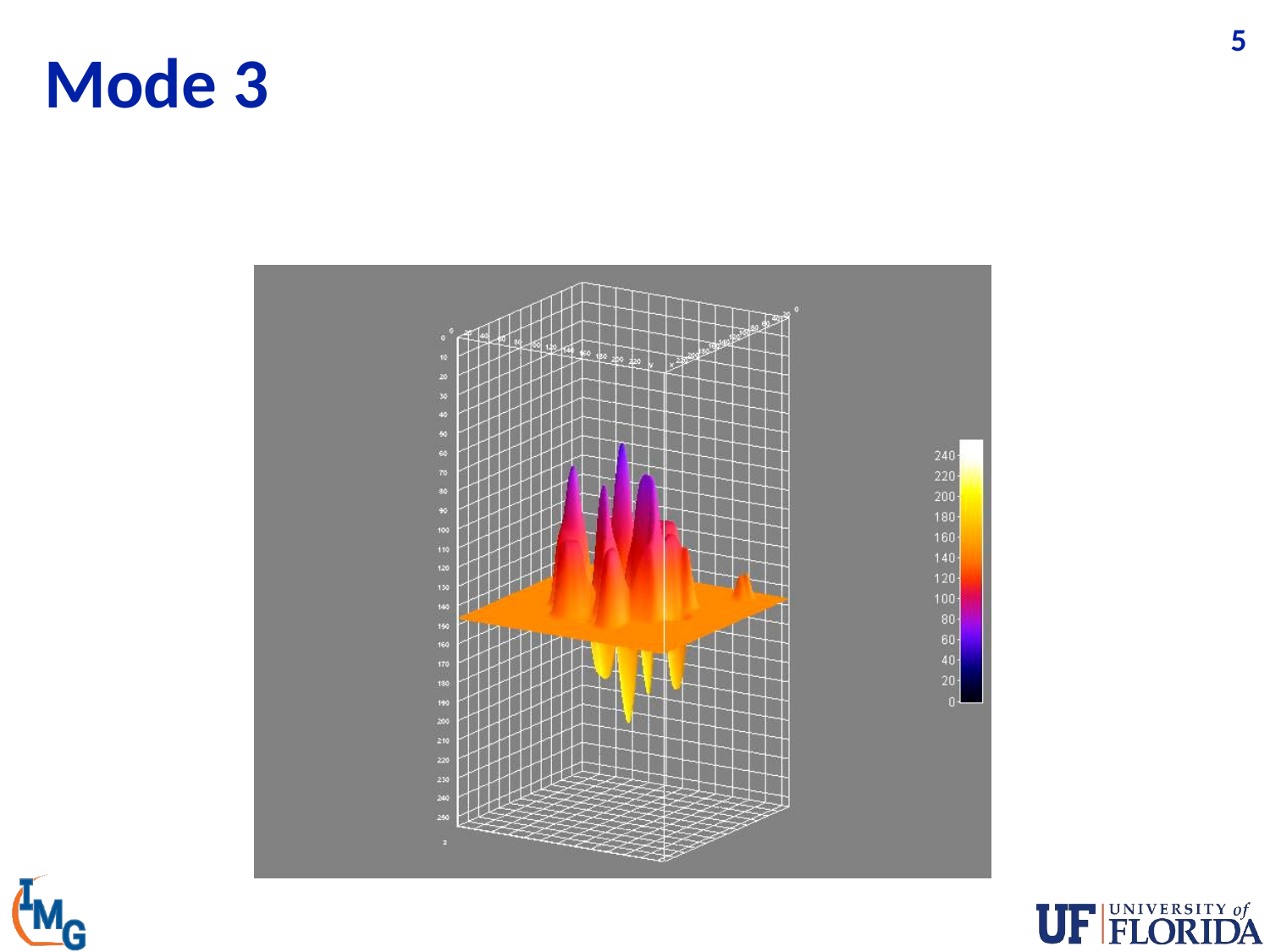

# Mode 3
4

## Slide 6
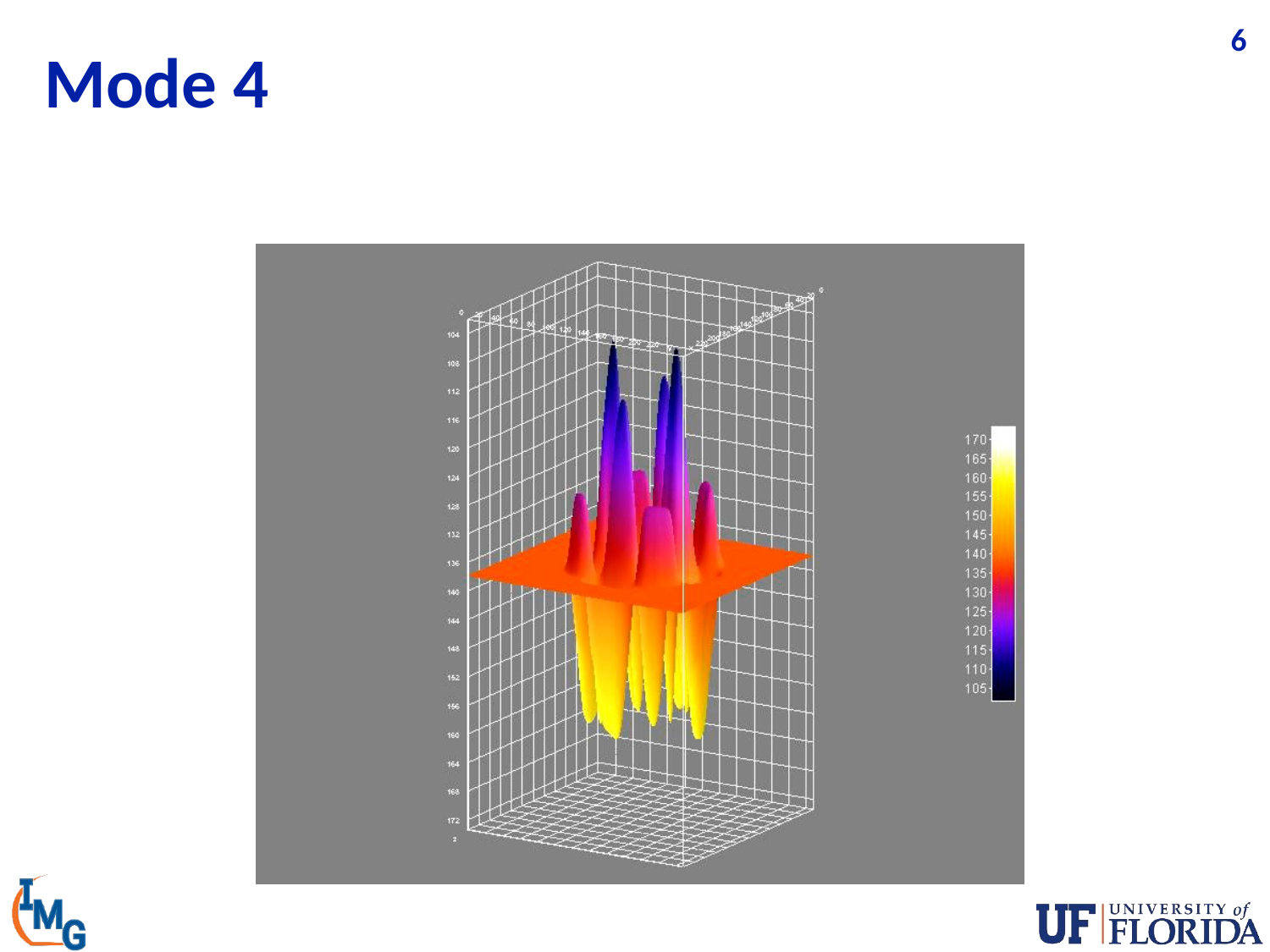

# Mode 4
5

## Slide 7
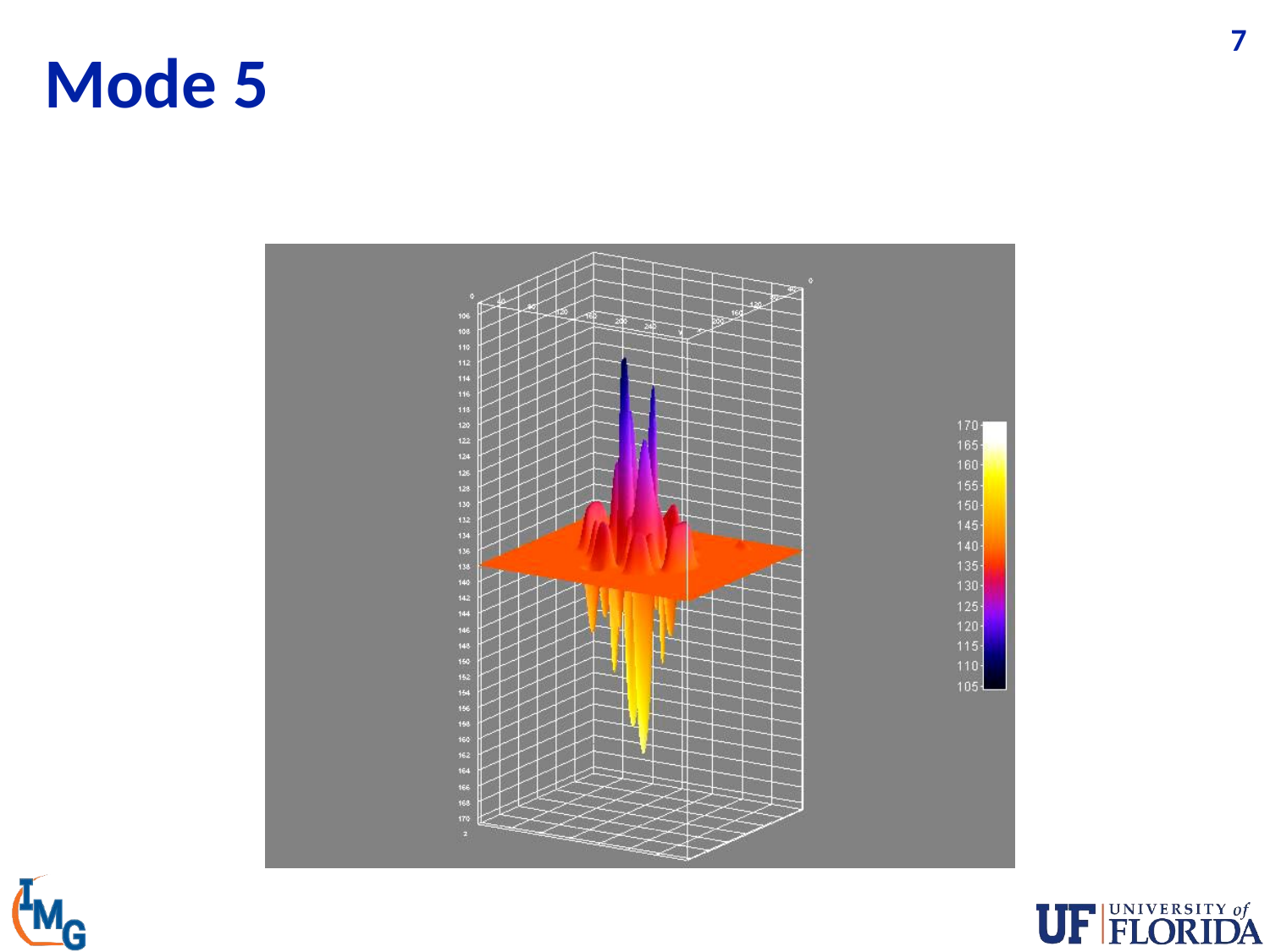

# Mode 5
6

## Slide 8
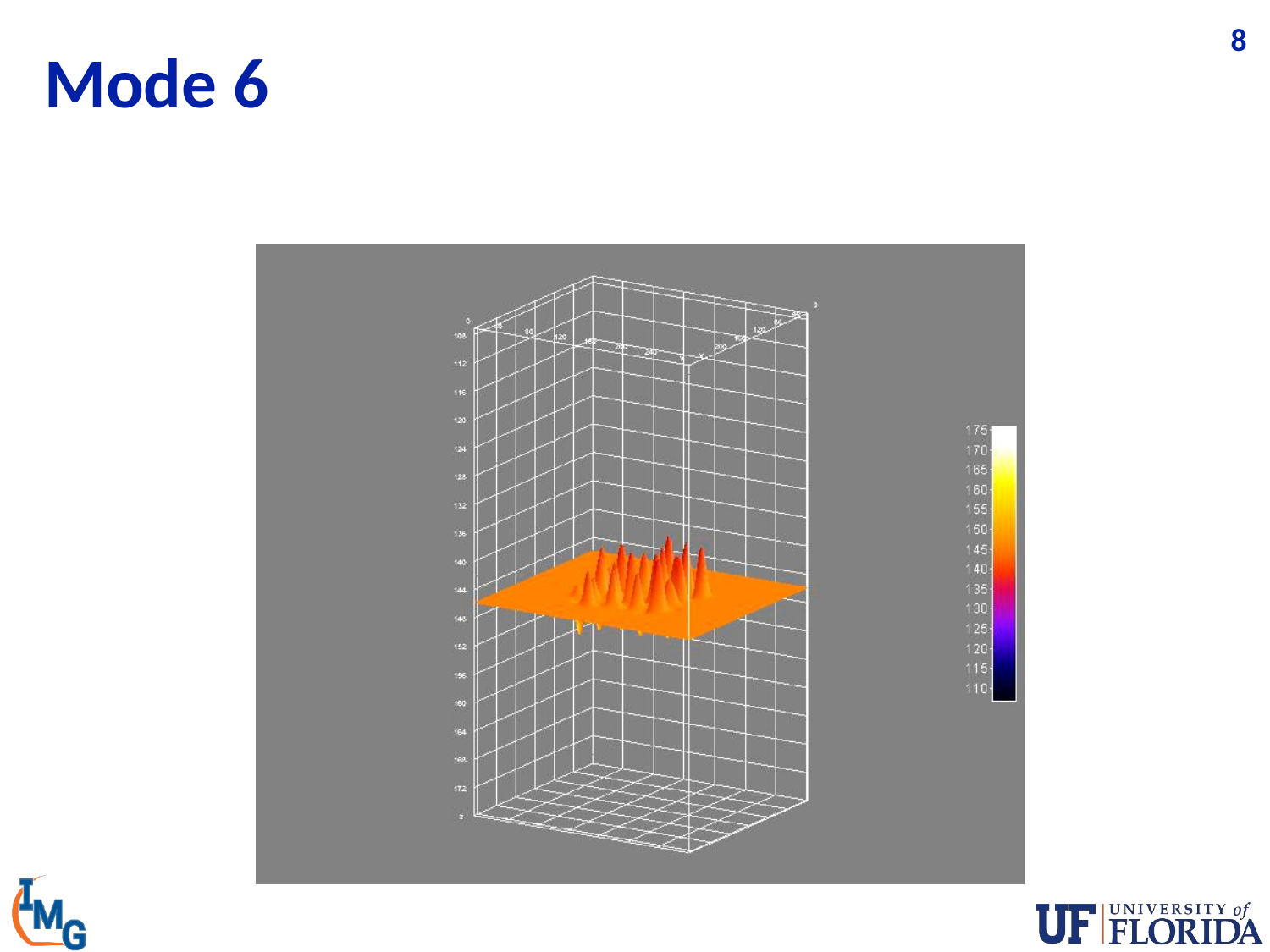

# Mode 6
7

## Slide 9
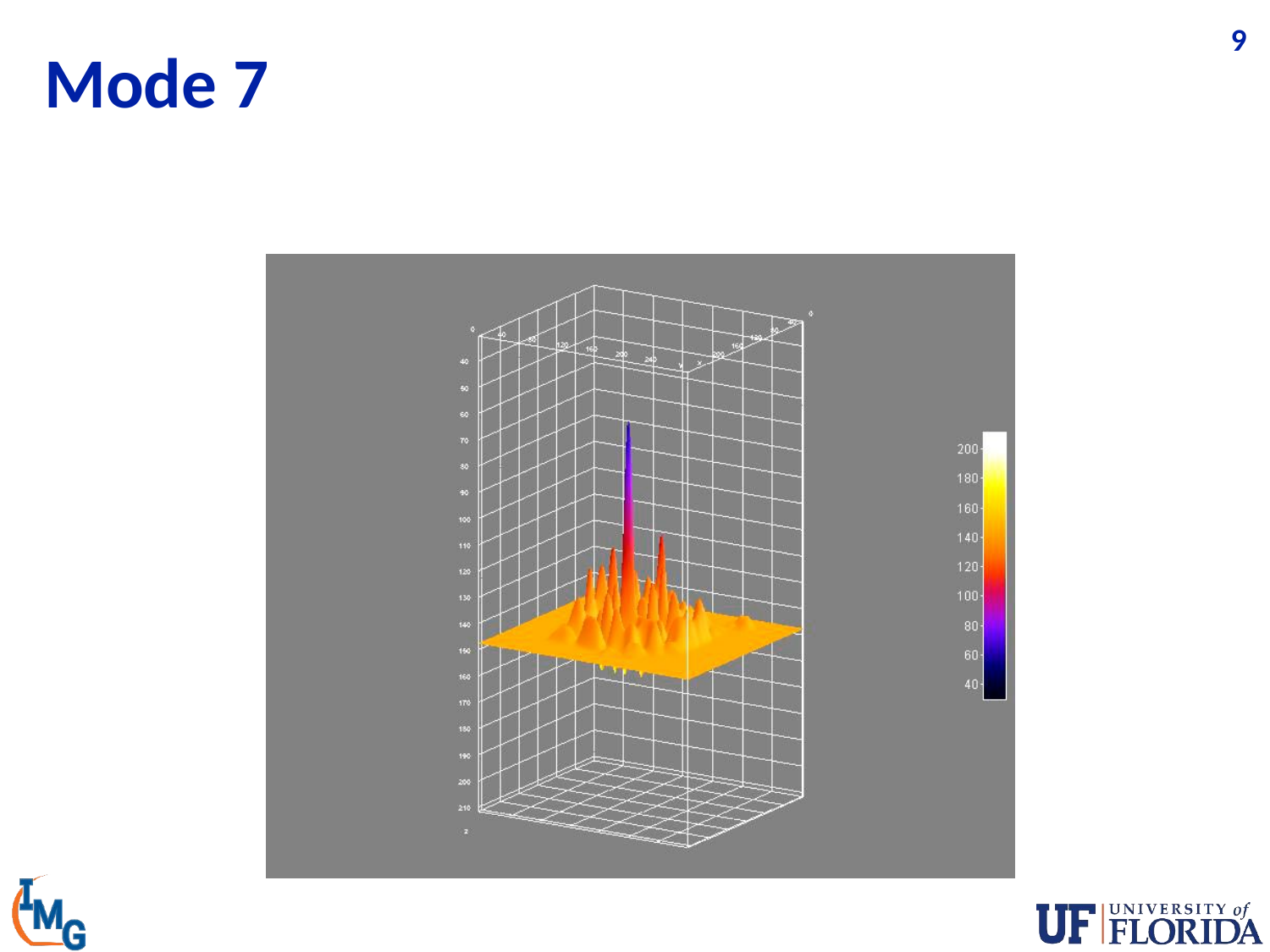

# Mode 7
8

## Slide 10
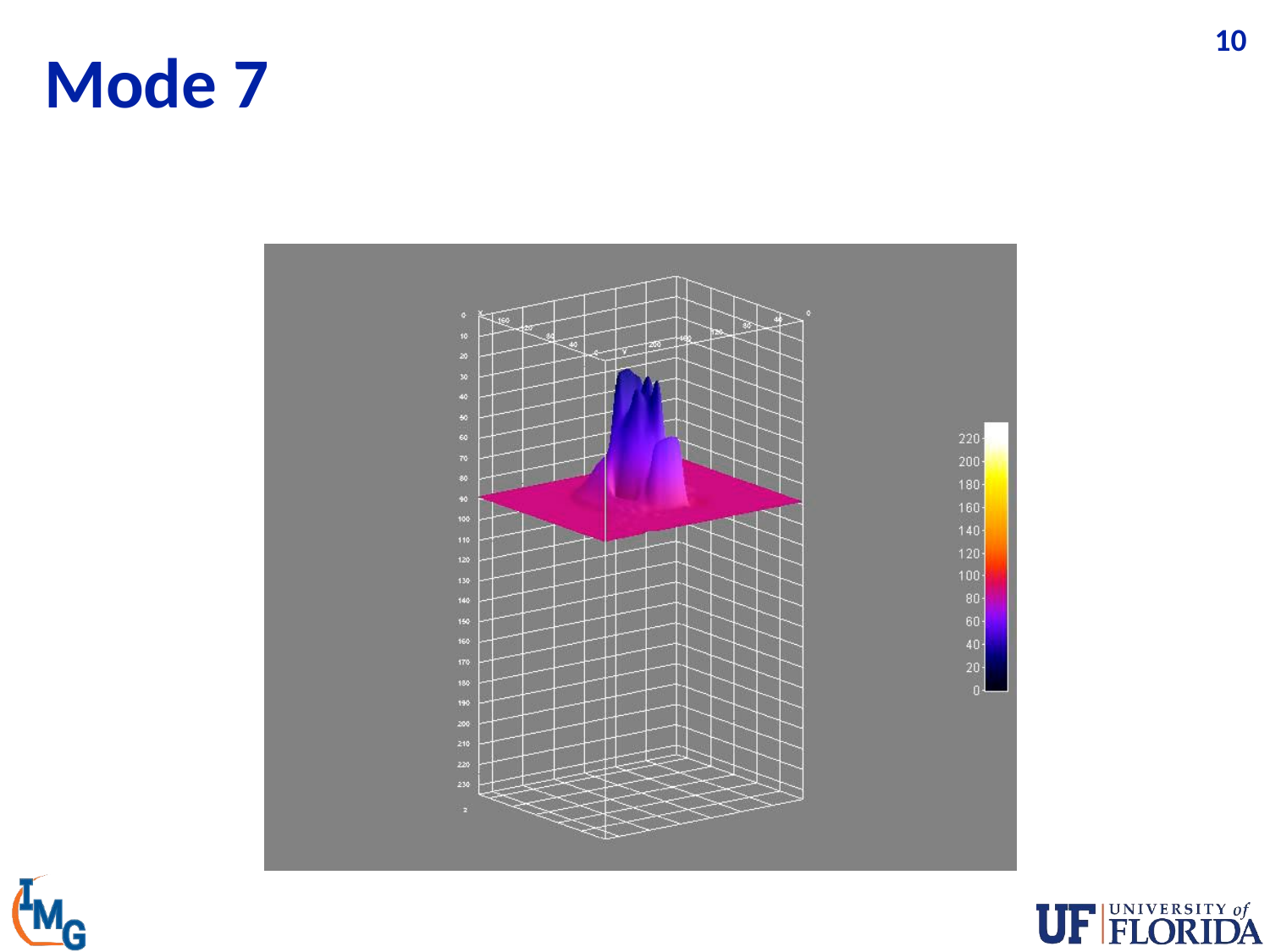

# Mode 7
9

## Slide 11
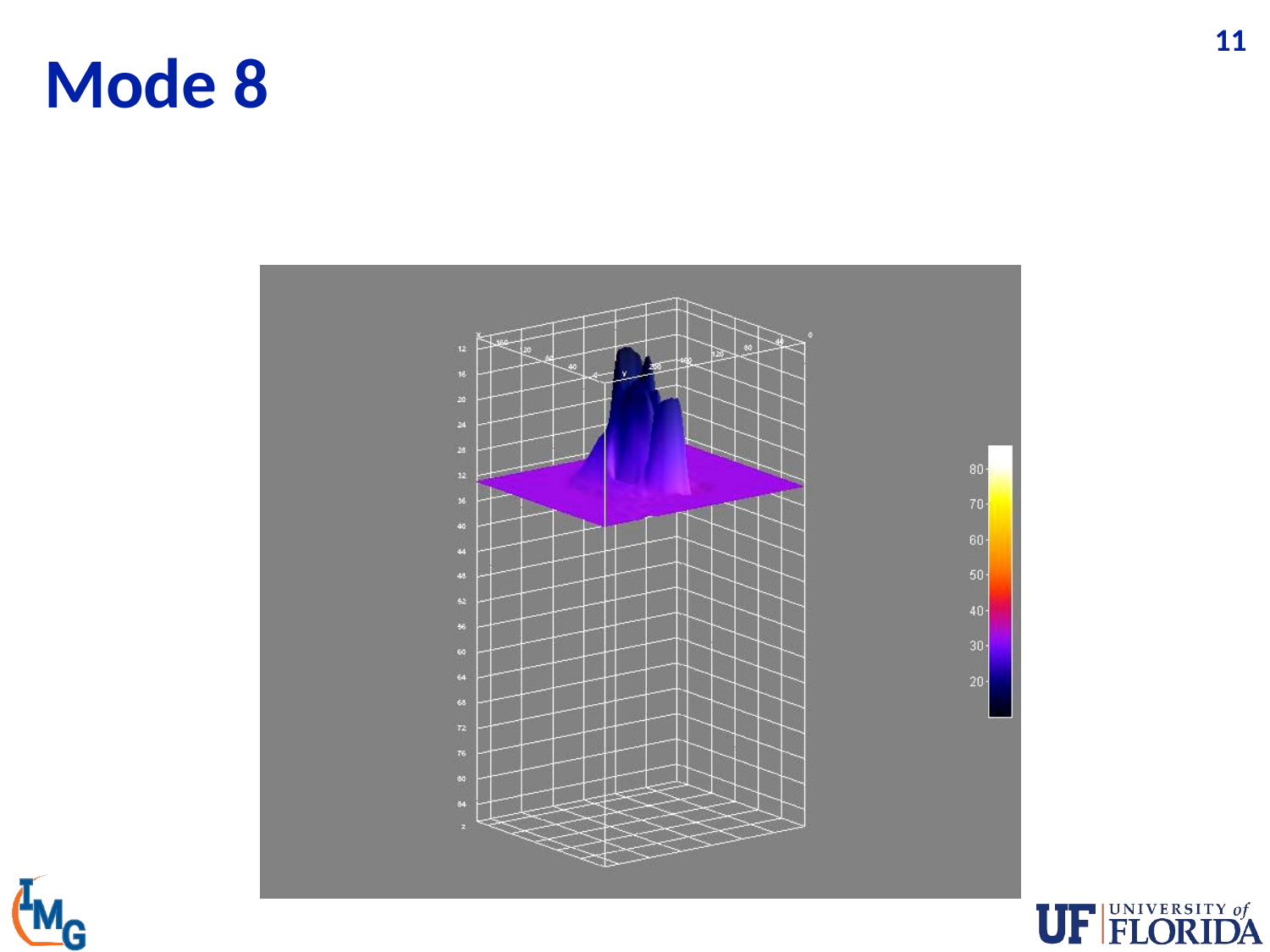

# Mode 8
10

## Slide 12
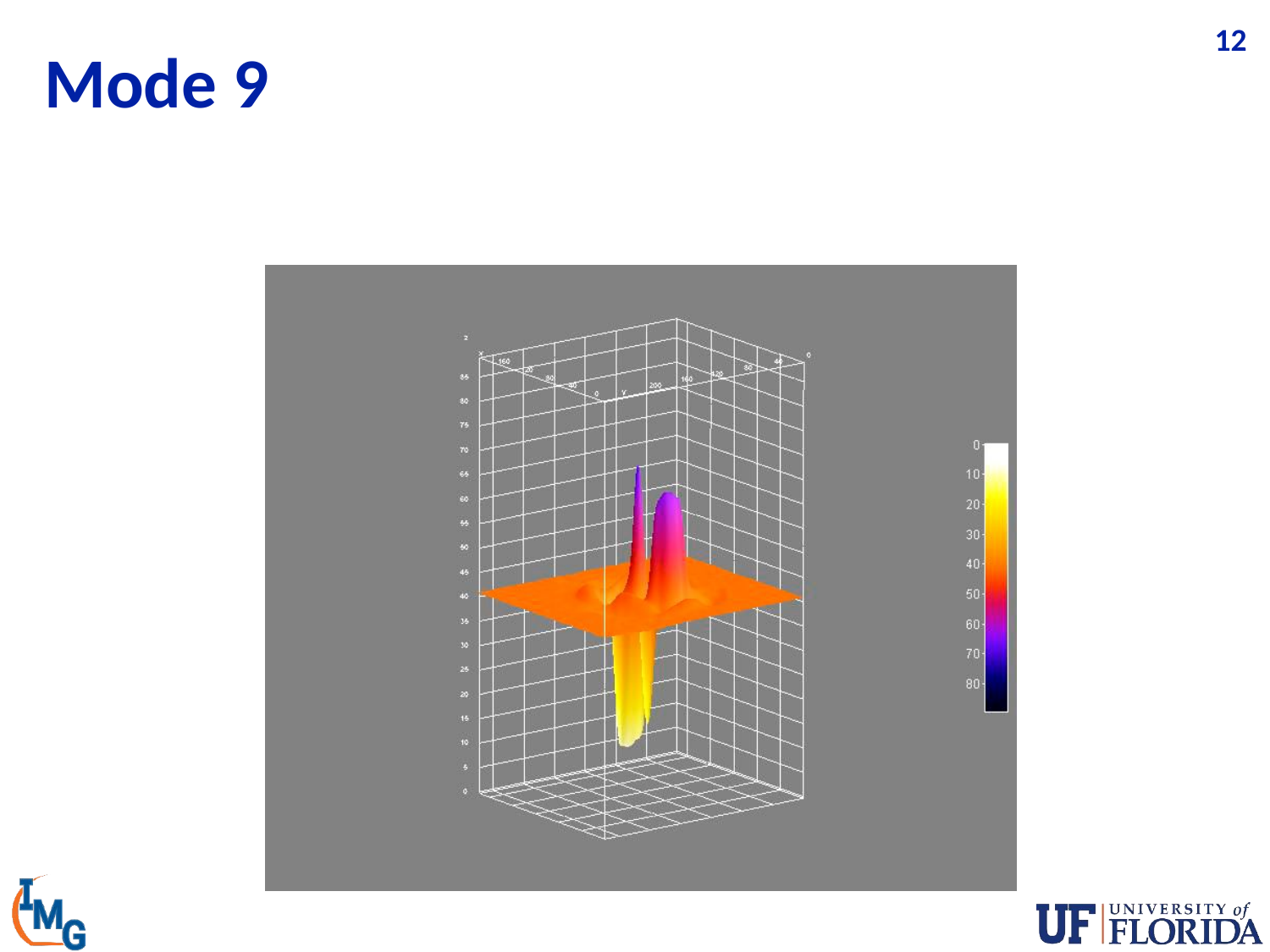

# Mode 9
11

## Slide 13
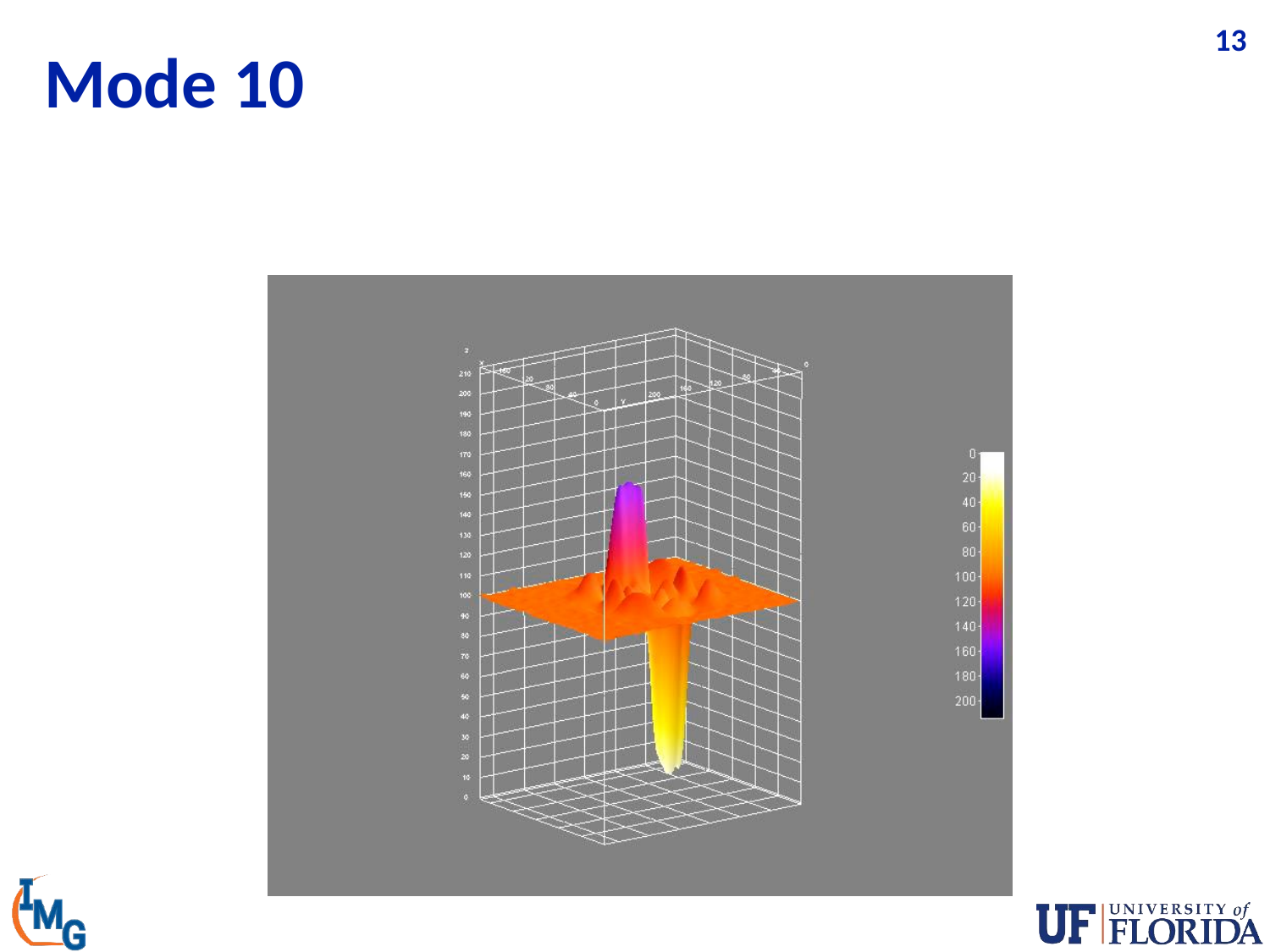

# Mode 10
12

## Slide 14
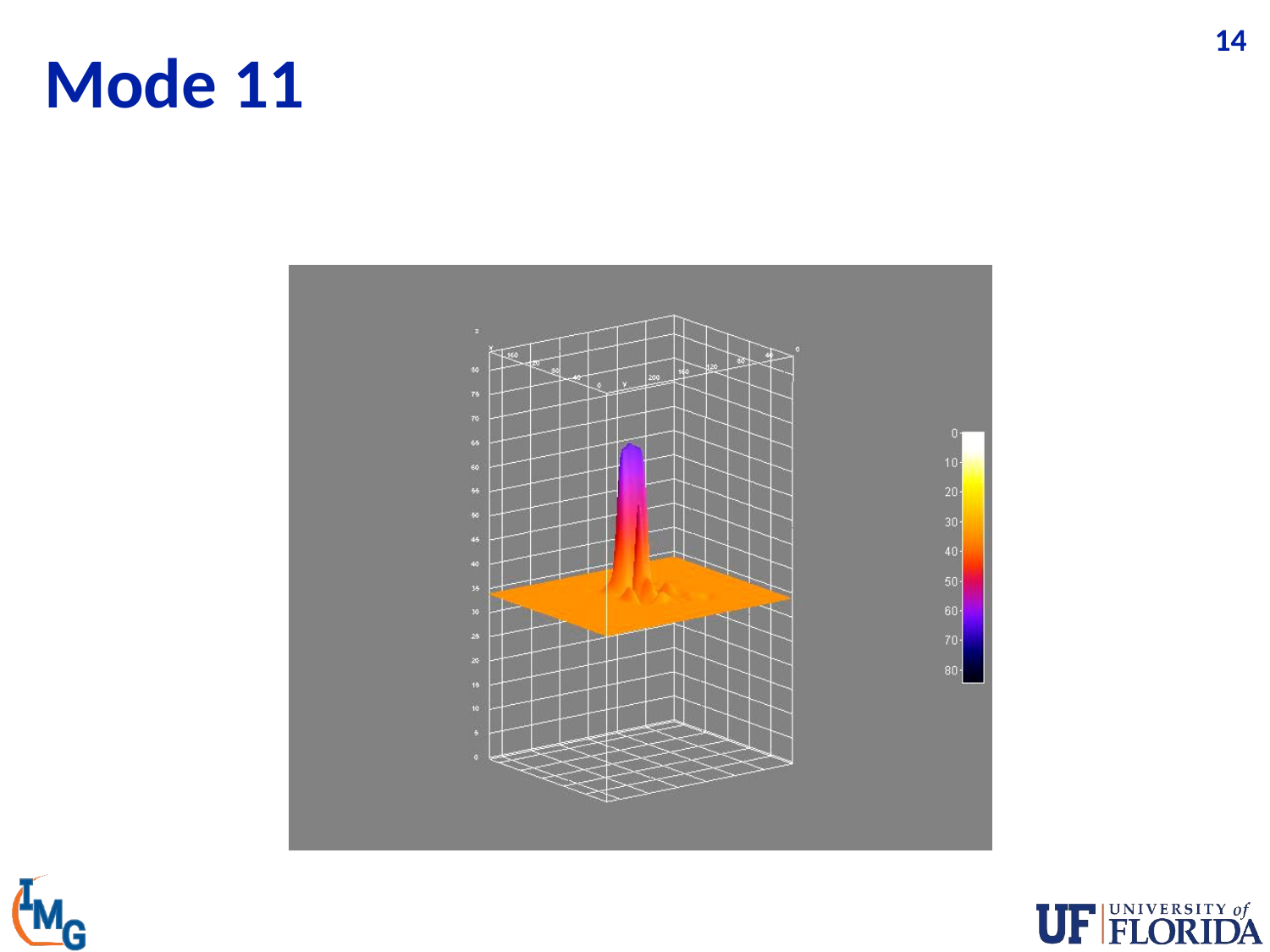

# Mode 11
13

## Slide 15
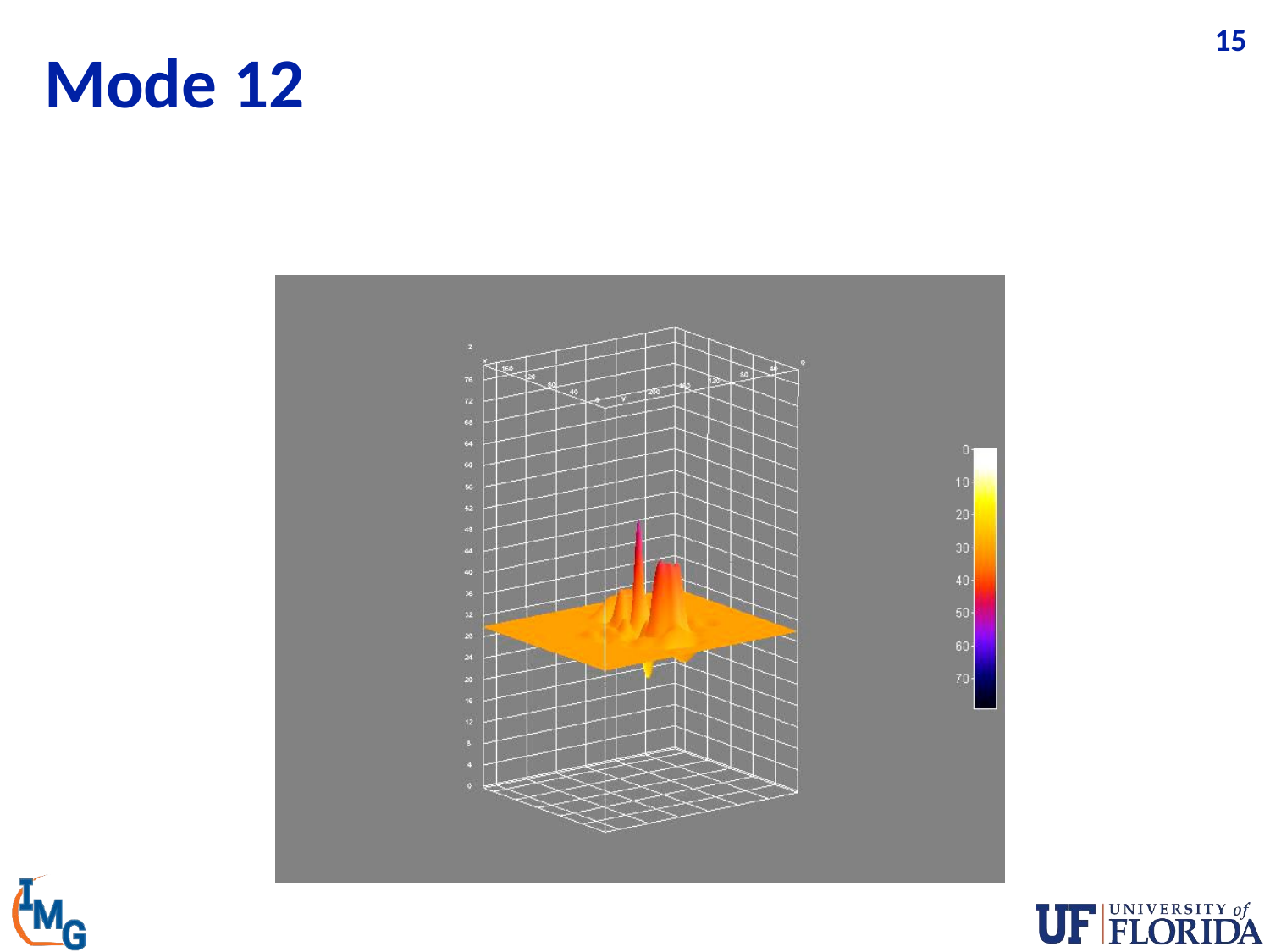

# Mode 12
14

## Slide 16
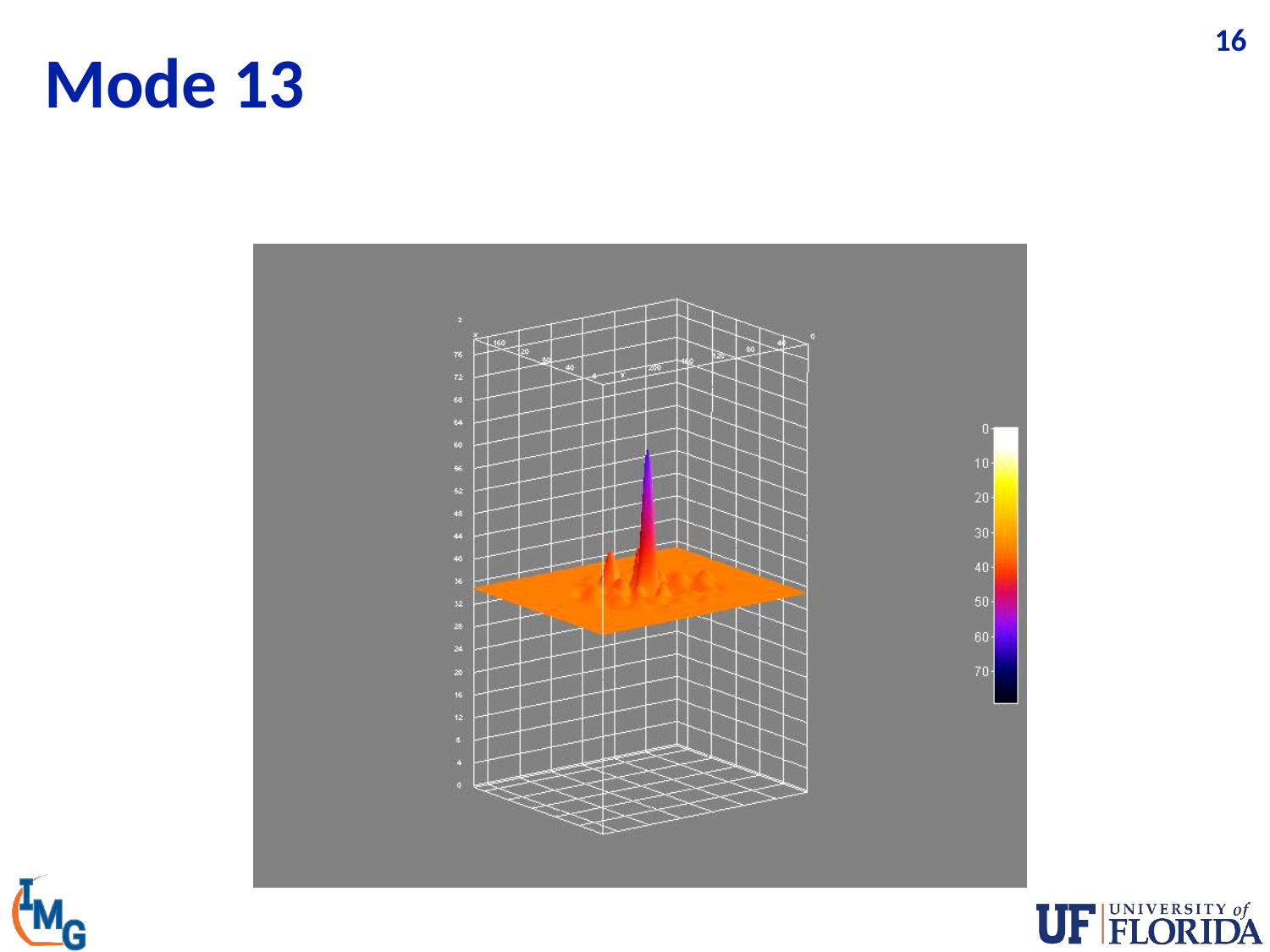

# Mode 13
15

## Slide 17
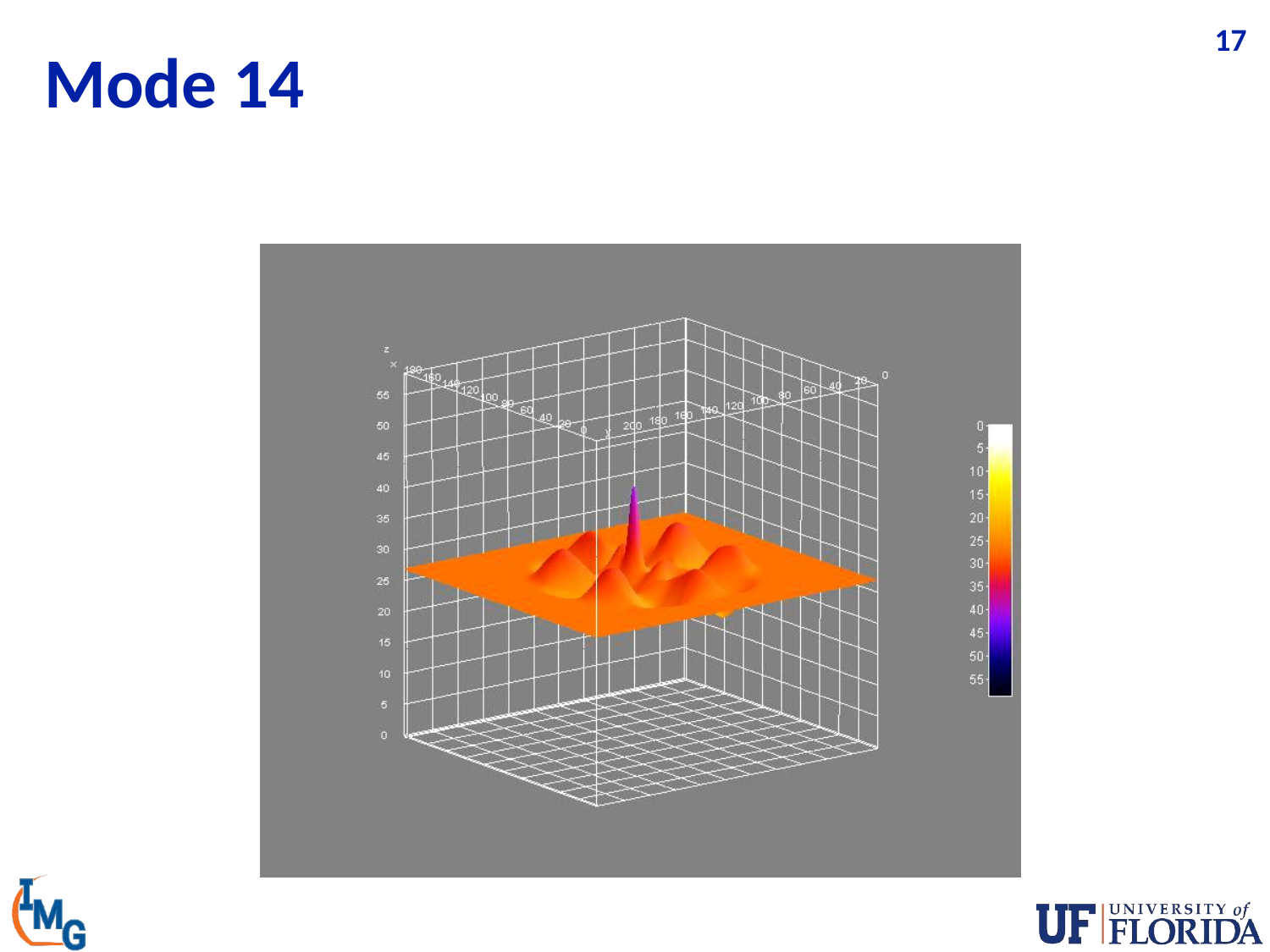

# Mode 14
16

## Slide 18
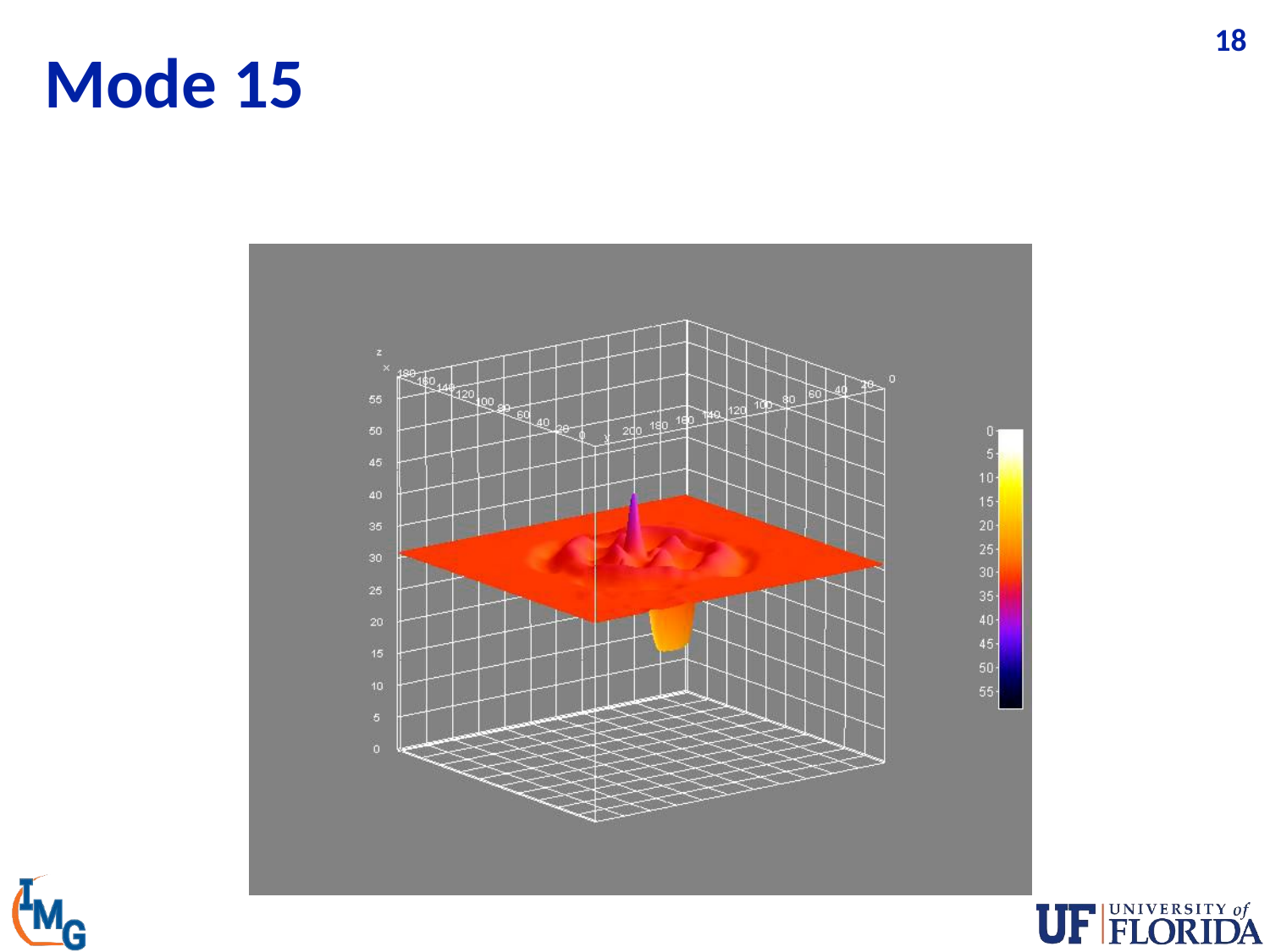

# Mode 15
17

## Slide 19
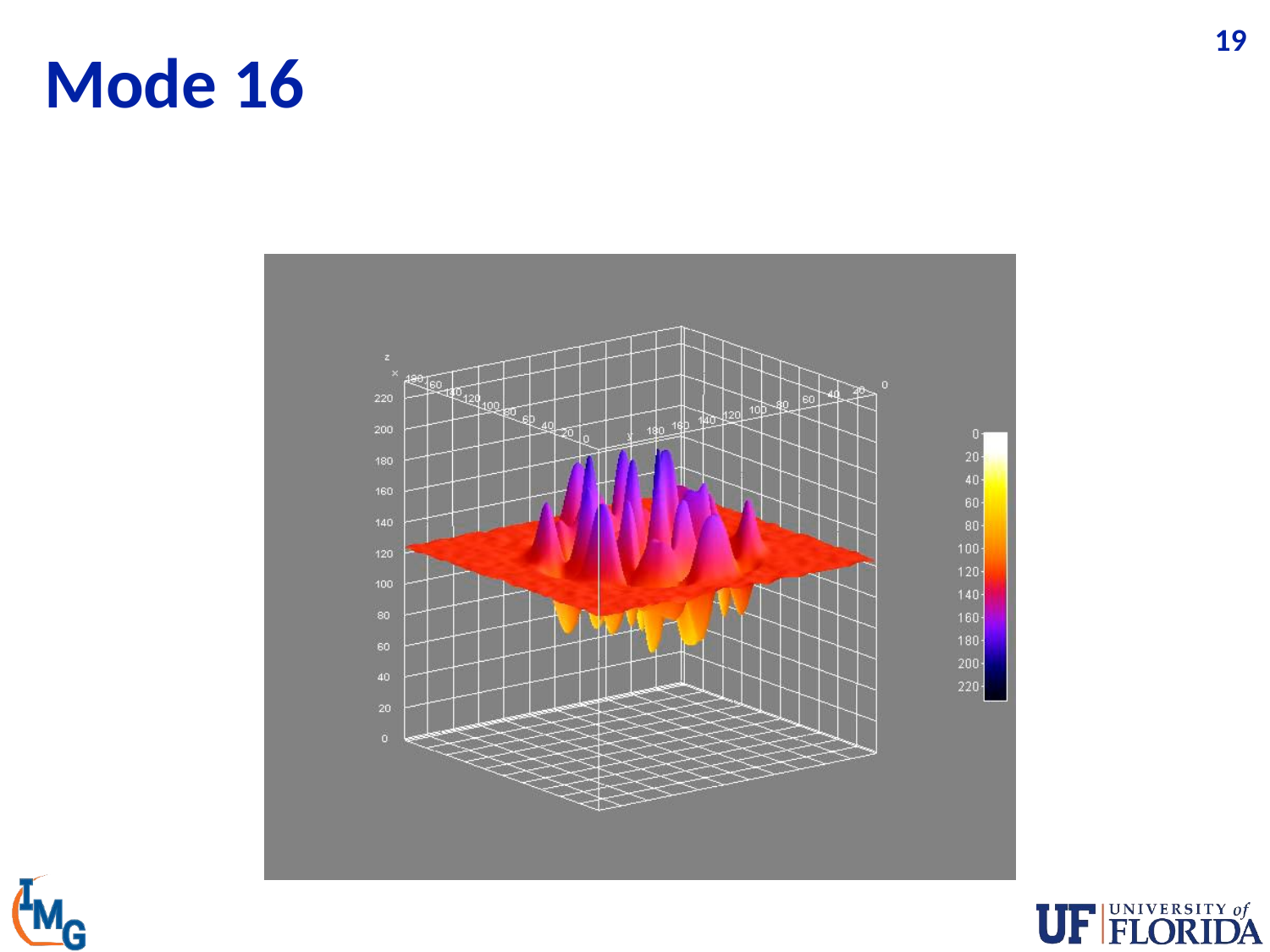

# Mode 16
18

## Slide 20
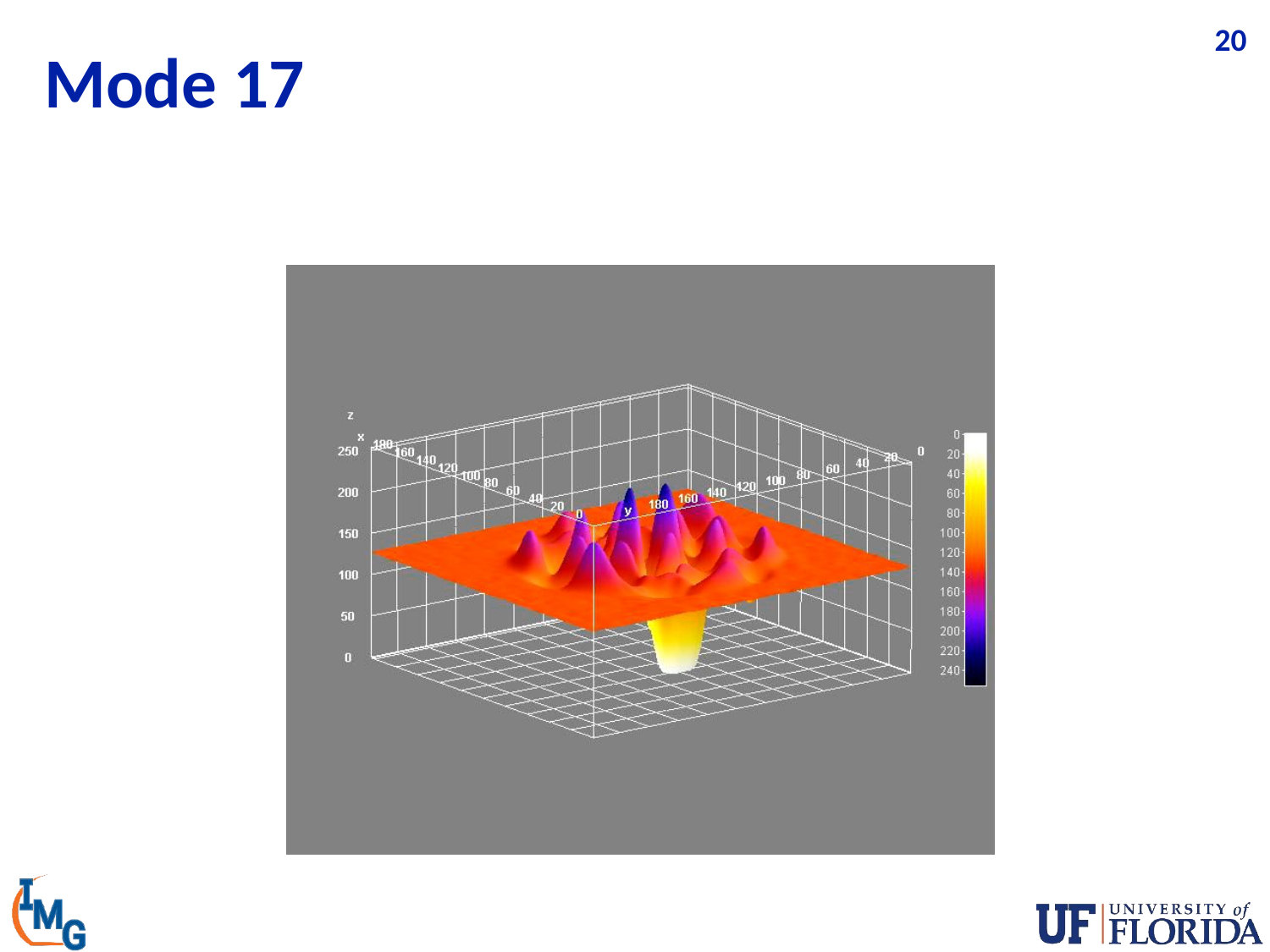

# Mode 17
19

## Slide 21
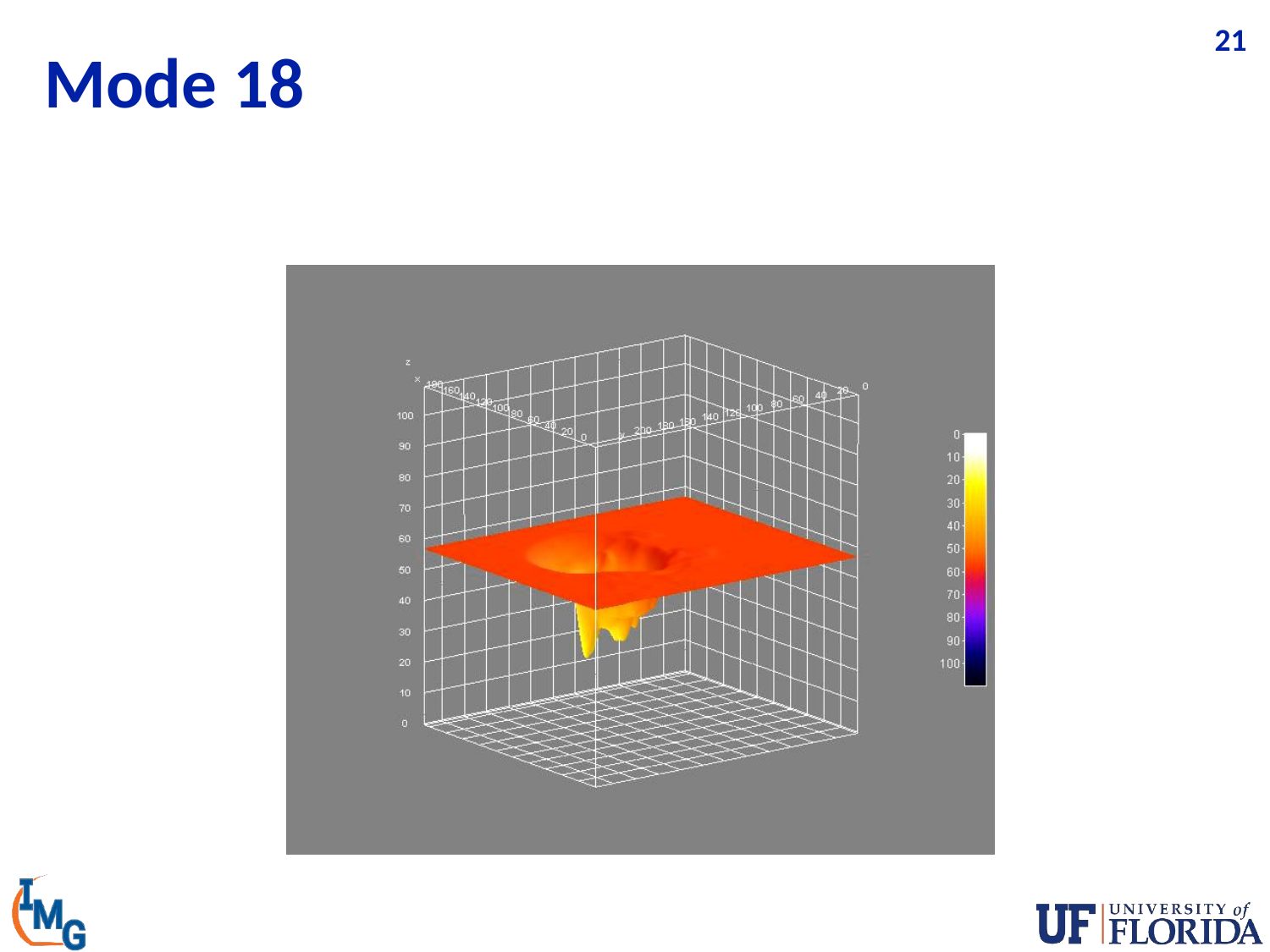

# Mode 18
20

## Slide 22
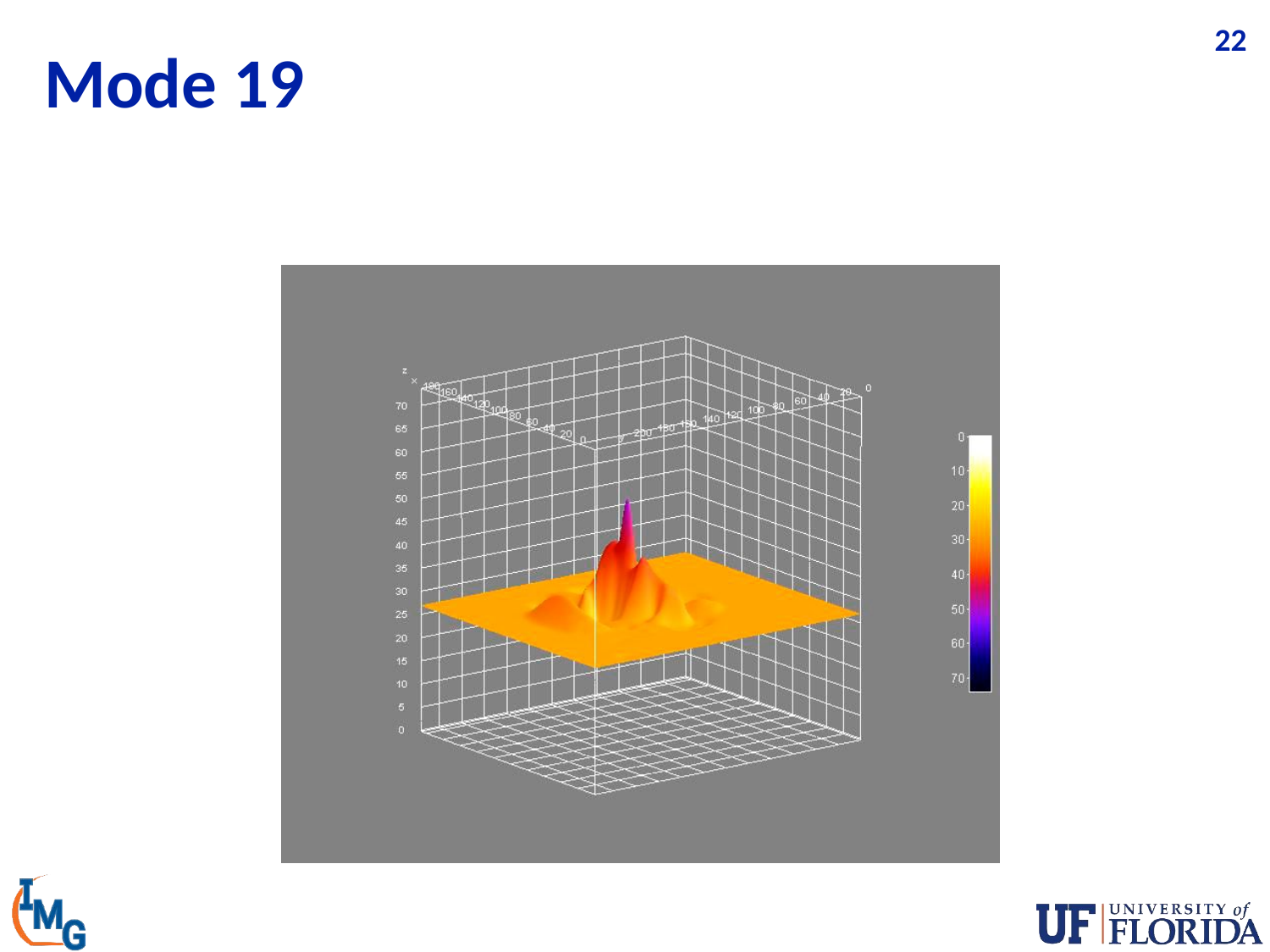

# Mode 19
21

## Slide 23
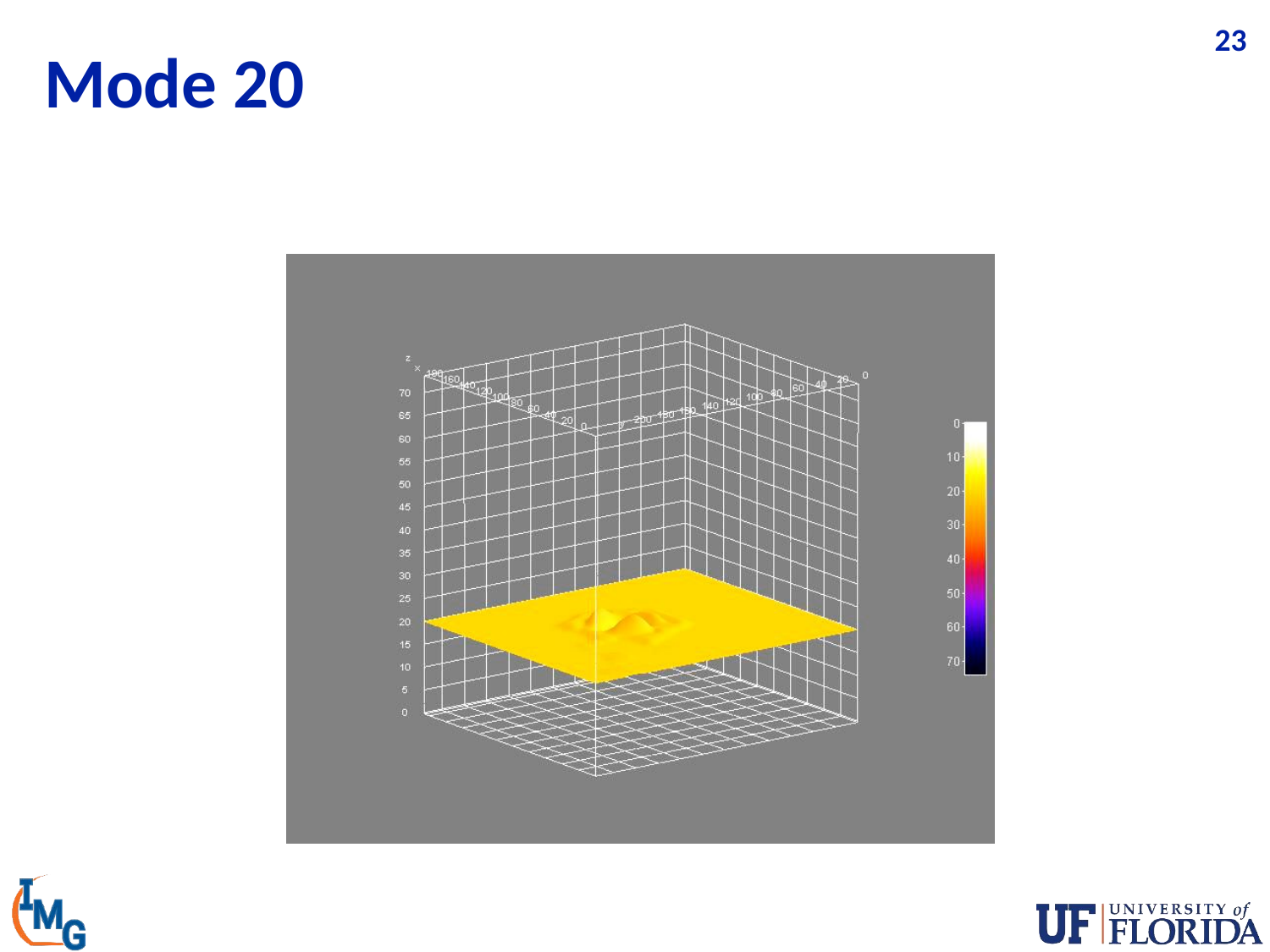

# Mode 20
22

## Slide 24
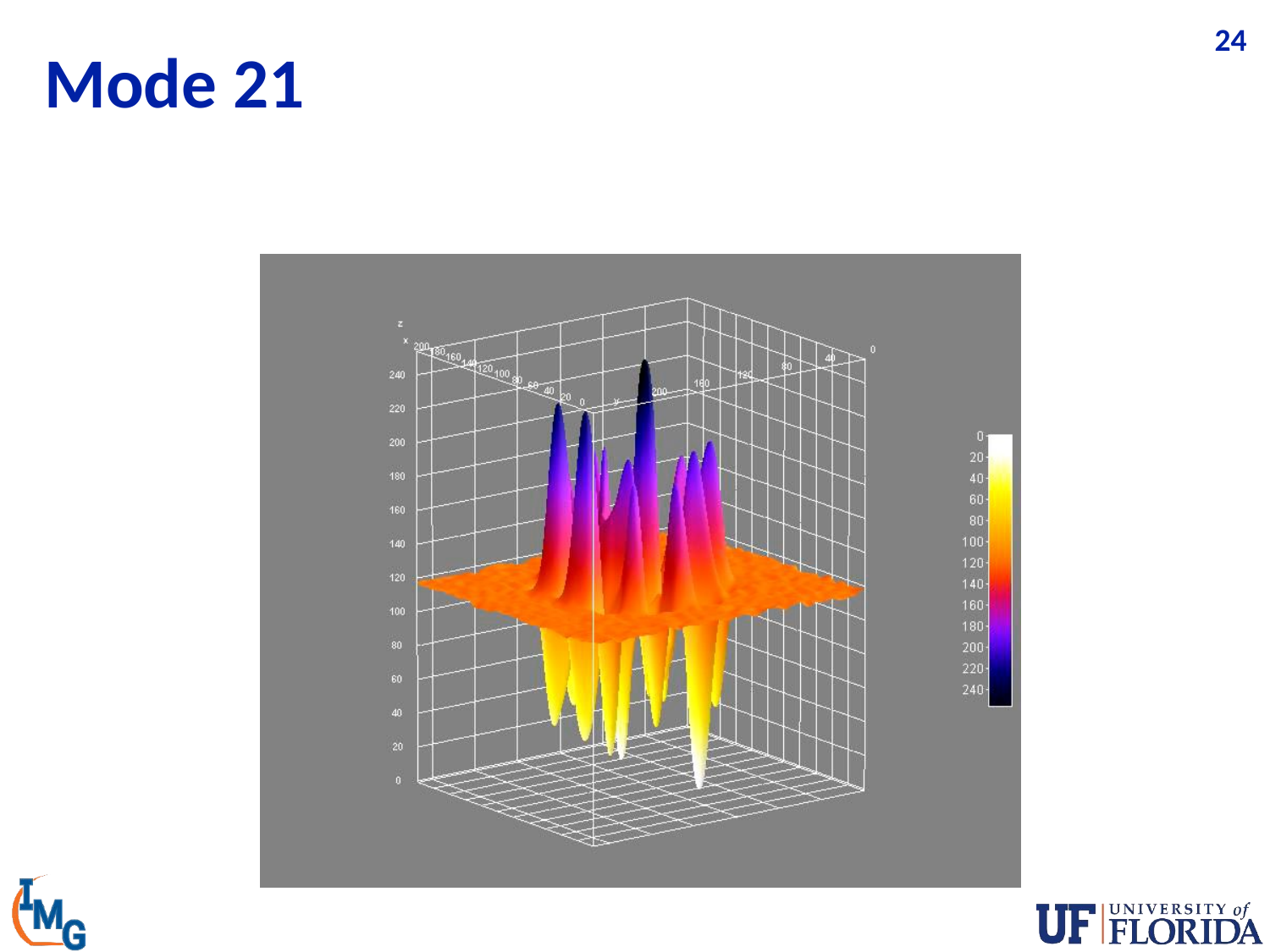

# Mode 21
23

## Slide 25
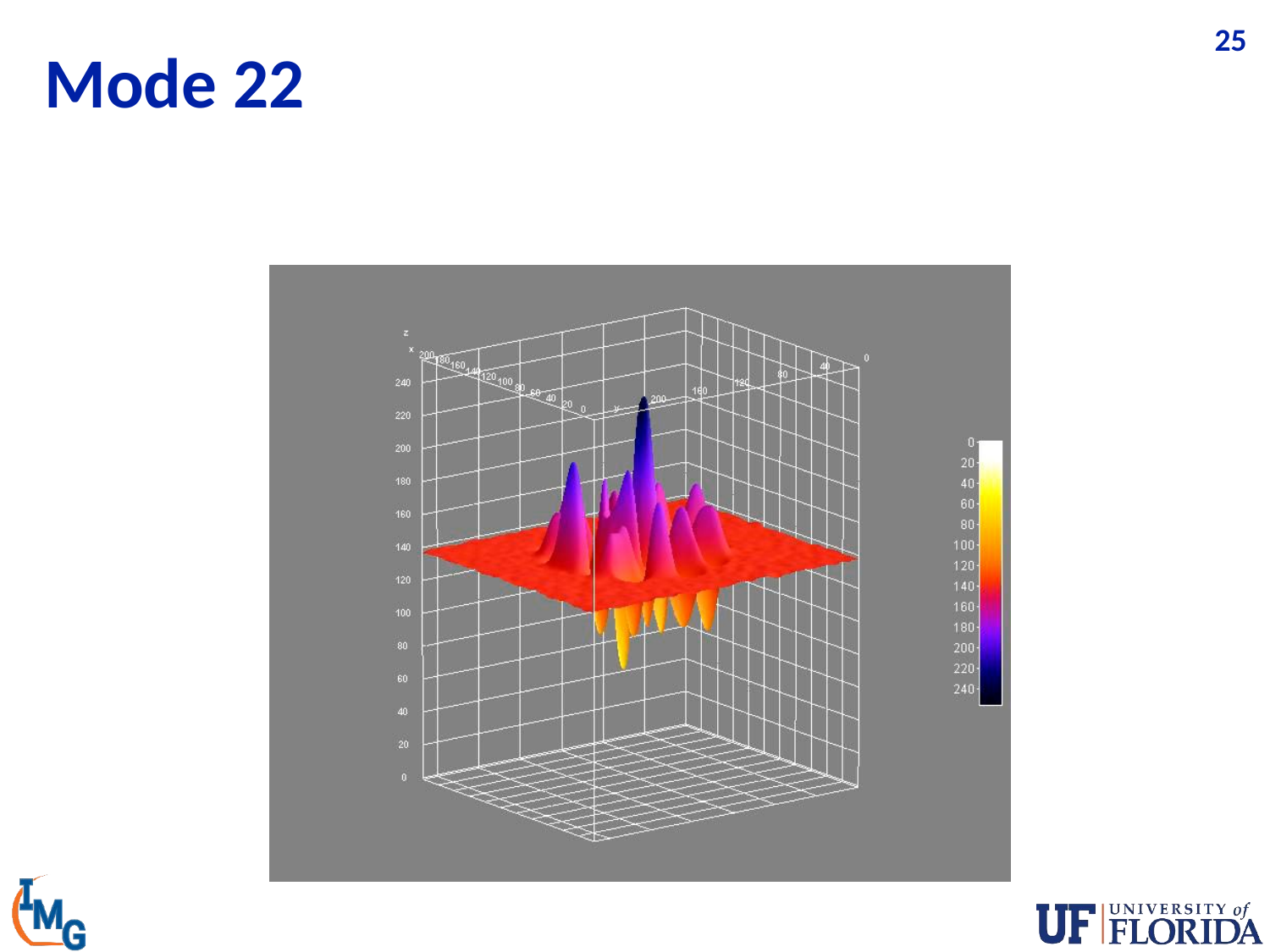

# Mode 22
24

## Slide 26
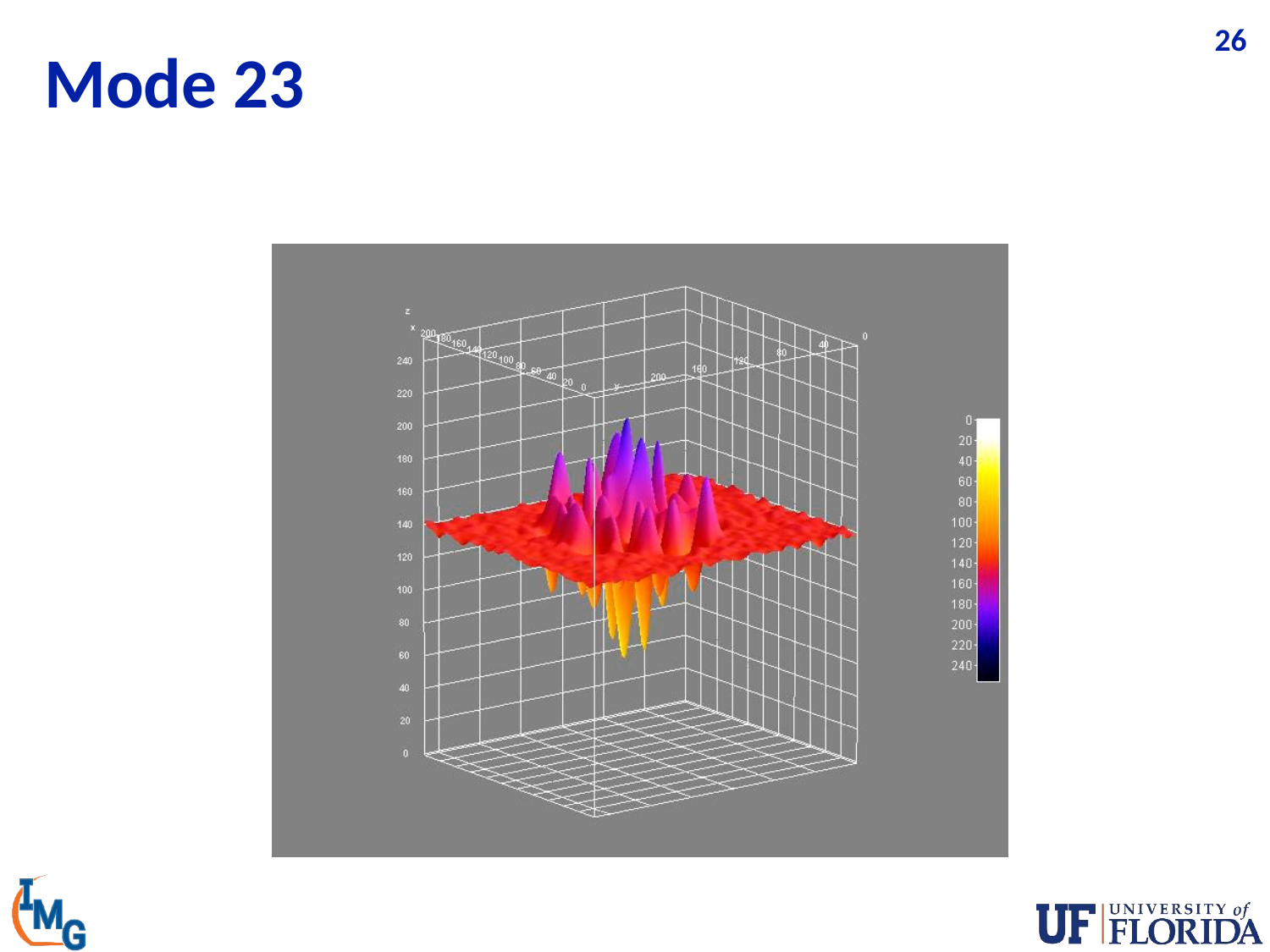

# Mode 23
25
